# Beyond the billion: dose-response immunophenotyping and machine learning classification of live vs. heat-treated gram-positive probiotic strains in human peripheral blood mononuclear cells—a single-donor proof-of-concept study

**DOI:** 10.3389/fmicb.2026.1943891

**Published:** 2026-10-26

**Authors:** Giovanni Deusebio, Annalisa Visciglia, Angela Amoruso, Marco Pane

**Affiliations:** Probiotical Research Srl, Novara, Italy

**Keywords:** cytokine profiling, dose-response, immunomodulation, machine learning, PBMC (peripheral blood mononuclear cells), postbiotic, probiotics

## Abstract

Probiotic research is constrained by three pervasive yet insufficiently challenged assumptions: the requirement for a minimum of 1 billion colony-forming units for efficacy, the necessity for gut colonization, and the inherent superiority of live over heat-treated preparations. This study addresses these gaps through a fully factorial experimental design evaluating 10 Gram-positive probiotic strains in both viable (Active Fluorescent Units, AFU) and heat-treated (Total Fluorescent Units, TFU) forms across three flow cytometry-verified concentrations (10^5^, 10^6^, and 10^7^ cells/well per ISO 19344:2015) in primary human peripheral blood mononuclear cells (PBMC) from a single healthy male Caucasian donor (58 years), with simultaneous quantification of 17 cytokines by BioPlex suspension array. Viable preparations induced substantially greater absolute cytokine responses than heat-treated preparations across 16 of 17 analytes, heat-treated preparations demonstrated stronger dose-response correlations (mean within-strain Spearman ρ up to 1.00) for 13 of 17 cytokines, a finding we attribute to the uncontrolled proliferation of live bacteria during 24-h co-culture compressing the effective concentration range. Six of ten viable strains exhibited monotonically increasing profiles; two strains displayed non-monotonic bell-shaped kinetics with peak activity at 10^6^ AFU/well and significant attenuation at 10^7^, directly falsifying the assumption that dose escalation uniformly increases immunological activity. MCP-1 was the sole cytokine showing no significant difference between viability states under the paired analysis (*q* = 0.98, geometric-mean ratio 0.99, 95% CI 0.89–1.12, excluding any difference beyond approximately 11% in either direction), providing an internal methodological control. In this single-donor model, unsupervised hierarchical clustering identified three immunological phenotype clusters, requiring multi-donor validation before these groupings can be treated as generalizable biological phenotypes, with Random Forest classification achieving 83.3% internal partition-recovery consistency (95% CI 66.4–92.7%; clusters derived from the same data; not an estimate of generalization to novel strains) vs. 33.3% chance; In this single-donor experiment, IL-12p70, IL-13, and IFN-γ, not IL-6 or IL-1β, were the primary discriminators of strain identity; generalizability of this ranking requires multi-donor validation. Heat-treated preparations achieved ≥70% functional equivalence relative to viable preparations at 10^7^ TFU/well in 80 of the 140 evaluable strain × analyte combinations, 57.1% of those evaluable (Functional Equivalence Dose, FED^70^), while one strain showed strongly attenuated activity in heat-treated form across all concentrations, a finding subject to the caveat that no positive control stimulus was included to formally verify PBMC functional competence on the experimental day. These findings establish a methodological framework integrating flow cytometric standardization, multiplex immunophenotyping, and machine learning for evidence-based dose characterization, postbiotic functional equivalence assessment, and data-driven strain classification in probiotic research (all *p*-values are descriptive within a single-donor experimental context).

## 1 Introduction

### 1.1 Cell model: rationale, validation, and analytical gaps

The rationale for the PBMC single-donor model, its analytical validation, and the specific analytical gaps addressed by this study are detailed in the Supplementary Material (Section S1).

This is a methodological study, not a functional evaluation of the strains it uses. It does not ask whether any of these preparations confers a health benefit, and no claim of probiotic or postbiotic efficacy is made or implied. It asks a prior set of questions that the field has left largely unanswered: whether a dose-response relationship can be demonstrated at all under controlled conditions, whether viable and heat-treated preparations of the same strain differ when supplied at matched concentrations, and what the limits of bacterial enumeration are when the two must be compared. This study was designed around four such questions, stated here before the analyses that address them. First, are there measurable differences in immunomodulation between viable and heat-treated preparations of the same strain at matched concentrations? Second, can dose-response relationships be demonstrated across a defined concentration range, and does their shape differ between strains? Third, how should bacterial doses be measured so that viable and heat-treated preparations are comparable? Fourth, can machine learning extract strain-level structure from multiplex cytokine data that univariate analysis does not reveal? The first three questions were specified before the experiment; the functional clustering and classification analyses that address the fourth were exploratory, and are identified as such where they are reported.

The formal recognition of this re-examination in the ISAPP postbiotic consensus is described in the Supplementary Material (Section S9).

The market context of these questions, the regulatory record on probiotic health claims and the enumeration crisis that motivates the choice of unit are set out in the Supplementary Material (Section S9).

## 2 Materials and methods

### 2.1 Study design and experimental overview

This study employed a fully factorial experimental design to evaluate the immunomodulatory properties of ten distinct Gram-positive probiotic bacterial strains on human peripheral blood mononuclear cells. The experimental factors comprised: bacterial strain identity (10 levels, designated A through L, excluding J and K); bacterial viability status (2 levels: live and heat-treated); and bacterial concentration (3 levels: 105, 106, and 107 AFU or TFU per well). Each experimental condition was assayed in technical triplicate, yielding a total of 180 treatment observations. Unstimulated peripheral blood mononuclear cell cultures served as negative controls (12 replicate wells); cytokine concentrations from the 12 wells were averaged per analyte prior to fold-change calculation, yielding a single negative control reference mean per cytokine used as the denominator for all conditions. The primary endpoint was the quantification of 17 cytokines in culture supernatants following 24-h co-incubation.

### 2.2 PBMC isolation and culture

Cryopreserved peripheral blood mononuclear cells were obtained from Lonza (Basel, Switzerland) as a pre-characterized preparation from a single healthy male Caucasian donor (58 years of age). Cells were thawed according to the manufacturer's protocol, washed twice with pre-warmed complete RPMI-1640 medium, and resuspended in complete medium supplemented with 10% heat-inactivated fetal bovine serum (FBS; Thermo Fisher Scientific, Waltham, MA, USA) and 2 mM L-glutamine.

### 2.3 Probiotic strain preparation

Ten probiotic strains (designated A–L for this analysis; full taxonomic characterization will be disclosed in subsequent publications) were maintained as cryostocks and revived by sequential subculturing. Each strain was grown in de Man–Rogosa–Sharpe (MRS) broth (Gibco, Thermo Fisher Scientific), supplemented with L-cysteine (1% v/v from a 5% stock) for the anaerobic strains, at its designated temperature and atmosphere (30° C or 37° C; aerobic or anaerobic in GasPak jars) as detailed in Supplementary Table 1; culture-collection deposit numbers will be added at proof stage. All strains were used at the second passage post-thaw to ensure consistent physiological state. For stimulation suspensions, strains were harvested from fresh broth culture at late-exponential phase, defined as a fresh overnight broth culture reaching a cell density of 1–3 × 10^9^ cells/mL, determined by flow cytometry, after the second passage, by centrifugation, washed once, and resuspended in sterile deionized water to eliminate potential confounding contributions of buffer components to PBMC cytokine responses.

#### 2.3.1 Thermal treatment protocol

Heat-treated (HT) preparations were produced from the same washed bacterial suspensions as viable preparations. A standardized thermal treatment protocol was applied identically across all 10 strains: exposure to a target temperature of 85° C for 60 min in a water bath, with temperature verified in the liquid phase, using 10 mL of suspension in 15 mL conical (Falcon) tubes; the heating ramp and cooling rate were not separately recorded, and suspensions were returned to room temperature on the bench. Complete treatment was confirmed by plating on appropriate solid recovery medium and confirming absence of colony growth after 48–72 h at optimal temperature for every batch and strain.

#### 2.3.2 Flow cytometric enumeration: AFU and TFU standardization

Quantification of both viable and heat-treated suspensions was performed by flow cytometry per ISO 19344:2015 using a NovoCyte flow cytometer (Agilent Technologies, Santa Clara, CA, USA) and Thiazole Orange (TO)/Propidium Iodide (PI) dual-fluorochrome staining. TO labels all bacteria regardless of membrane integrity; PI selectively penetrates membrane-compromised cells. Viable bacteria (TO^+^/PI^−^) were quantified as Active Fluorescent Units per well (AFU/well); heat-treated bacteria (TO^+^/PI^+^) as Total Fluorescent Units per well (TFU/well). This approach, validated for heat-treated bacterial cells using TO/PI staining (PMC11099204, 2024), enables direct, concentration-matched comparison between viable and heat-treated preparations in identical physical units (cells per well), independently of culturability—resolving the fundamental CFU limitation identified by Davis (2014), Beal et al. (2020), and Foglia and Allesina (2020). Final concentrations of 10^5^, 10^6^, and 10^7^ AFU/well or TFU/well were confirmed by post-dilution re-measurement prior to addition to PBMC cultures.

### 2.4 PBMC-bacteria co-culture

Peripheral blood mononuclear cells were seeded in 48-well flat-bottom plates at a density of 1 × 106 cells per well in 1 mL antibiotic-free RPMI-1640 medium supplemented with 10% heat-inactivated fetal bovine serum and 2 mM L-glutamine. Bacterial preparations (live or heat-treated) were added at the designated concentrations. Co-cultures were incubated at 37° C in a humidified atmosphere containing 5% CO_2_ for 24 h. Following incubation, culture plates were centrifuged at 300 × g for 10 min, and cell-free supernatants were carefully collected and stored at −80° C until cytokine quantification.

### 2.5 Multiplex cytokine quantification

Cytokine concentrations in culture supernatants were quantified using the Bio-Plex Pro Human Cytokine 17-plex Assay (Bio-Rad Laboratories, Hercules, CA, USA) according to the manufacturer's instructions. The panel comprised: G-CSF, GM-CSF, IFN-γ, IL-1β, IL-2, IL-4, IL-5, IL-6, IL-7, IL-8/CXCL8, IL-10, IL-12p70, IL-13, IL-17A, MCP-1/CCL2, MIP-1β/CCL4, and TNF-α. Eight-point standard curves were generated for each cytokine, and concentrations were calculated by 5-parameter logistic regression curve fitting. Quality control criteria included intra-assay CV ≤ 15% and inter-assay CV ≤ 20%.

### 2.6 Data analysis and statistics

Strain identities were coded A–L before any analysis was undertaken. The investigator performing the statistical and machine-learning analysis worked from these codes alone; the key linking each code to its species and in-house designation was held by a separate investigator and was not consulted while the analyses reported here were being run. The functional groupings, the classification results and the dose-response classifications were therefore obtained without knowledge of which species each code represented, and no analysis was selected or repeated on the basis of which strain produced which result. Immunomodulatory activity was expressed as fold-change (FC) relative to mean cytokine concentration in unstimulated control wells of the same experimental run. Data are presented as mean ± SD across three technical replicates. The negative control denominator represents the intra-analyte mean of 12 replicate wells. Consequently, fold-change SD values reported for each condition reflect within-condition technical variability only, and do not capture uncertainty in the negative control reference value.

#### 2.6.1 Data preprocessing

Out-of-range values below the detection limit were replaced with the minimum concentration observed for that analyte across all experimental samples in the dataset. Out-of-range values exceeding the upper quantification limit were replaced with the maximum concentration observed for that analyte across all experimental samples in the dataset. This approach anchors substituted values to the empirically determined quantification boundaries of the specific assay run, avoiding arbitrary scalar multipliers. Log_2_-transformation, as declared in the deposited configuration file, was applied to normalize distributions and stabilize variance. For multivariate analyses, RobustScaler normalization (using median and interquartile range) was employed to reduce the influence of extreme outliers.

Three analyte subsets are used, and each analysis is assigned to one of them *a priori* in the deposited configuration file. (i) All 17 analytes are used for the fold-change, dose-response and Bayesian analyses, where each analyte is treated independently and quantification-limit saturation does not propagate between variables. (ii) Sixteen analytes, with IL-8 excluded, are used for all supervised classification: IL-8 exceeded the upper limit of quantification in the majority of viable conditions, so after imputation it behaves as a near-binary saturation flag rather than a graded biological signal, and a classifier trained on it learns the assay limit rather than the immunology. (iii) Fourteen analytes, with IL-8, MIP-1β, and TNF-α excluded, are used for the heatmaps, for principal component analysis and for the Functional Equivalence Dose matrix, because all three reached or exceeded the upper limit of quantification in a substantial proportion of conditions and their imputed values would dominate a variance-based decomposition. Subset membership is fixed in the configuration file and is not varied between analyses.

#### 2.6.2 Differential expression and dose-response analysis

Viable and heat-treated preparations were compared as matched pairs. Each viable condition has a natural counterpart at the same strain and the same concentration, so differences in cytokine expression between viability states were evaluated using the Wilcoxon signed-rank test on the 30 matched strain x concentration condition pairs (viable vs. heat-treated), with Benjamini-Hochberg false discovery rate correction across the 17 analytes (Benjamini and Hochberg, 1995). This replaces the unpaired Mann-Whitney U test used in the previous version of this analysis, which treated the two viability groups as independent and therefore did not match the paired architecture of the design. The unit of analysis is the condition mean (*n* = 30 pairs); technical replicates are averaged before testing and are not treated as independent observations. It is noted that the 30 condition means per viability group are not fully independent observations, as they derive from a single biological donor with *n* = 3 technical replicates per condition. Because all conditions derive from one donor, the *p*-values reported throughout this study should be interpreted as descriptive indicators of effect magnitude and direction within this experiment rather than as inferential probability statements generalizable to a broader population. All statistical comparisons are reported in this spirit—as structured descriptions of the observed data within this single-donor experiment, consistent with the exploratory and hypothesis-generating design of the study. Fold-change was calculated as the ratio of geometric means. Dose-response relationships were assessed using Spearman rank correlation between bacterial concentration and cytokine levels, stratified by viability status. Kruskal-Wallis tests were used to evaluate overall concentration effects within each viability state. Effect size for each paired comparison is reported as the geometric-mean ratio of the viable to the heat-treated fold-change across the 30 matched condition pairs, with a 95% confidence interval obtained on the log_2_ scale and back-transformed, together with the matched-pairs rank-biserial correlation, so that magnitude and its precision can both be assessed independently of significance. The geometric-mean ratio is used as the single effect measure throughout; ratios are computed on condition means rather than on individual replicates, so that the effect size and the significance test operate on the same unit of analysis. A pre-declared biological relevance threshold of 1.5-fold, the same criterion applied in the Bayesian analysis (Section 2.7.3), is used throughout to distinguish statistically detectable from biologically meaningful differences.

#### 2.6.3 Dimensionality reduction and clustering

Principal component analysis was performed on RobustScaler-standardized log-transformed data. Component retention was determined using the Kaiser criterion and scree plot inspection, targeting at least 80% cumulative variance explained. K-means clustering was applied to the full dataset with the optimal cluster number determined by maximizing both silhouette score and Calinski-Harabasz index across *k* = 2–10. Hierarchical clustering using Ward's minimum variance method with Euclidean distance provided complementary validation of the partition structure. PCA was applied at two levels: (1) on the full observation matrix (180 treatment observations × 17 cytokines) to characterize the global variance structure of the dataset including technical replicates, and (2) on the condition-mean matrix (60 conditions × 14 cytokines, means of technical replicates) to visualize the immunological space occupied by each strain × viability × dose combination and support hierarchical clustering of strain-level fingerprints.

#### 2.6.4 Software

All analyses were performed in Python 3.12 using scikit-learn (Pedregosa et al., 2011), scipy, pandas, xgboost, matplotlib, and seaborn. The complete analysis package—raw data, configuration file declaring every methodological choice and random seed, source code, canonical output values and a provenance ledger mapping each reported number to its recomputation—is deposited on Zenodo (DOI to be inserted at proof stage). A verification script re-runs the pipeline and asserts that every reported value reproduces; the computing environment is pinned and containerised.

*AI disclosure:* Generative artificial intelligence (Claude, Anthropic, and claude.ai) was used in the preparation of this manuscript for scientific writing and structural editing, Python scripting for figure generation and machine learning analysis, and literature synthesis. All AI-generated content was critically reviewed, verified against primary sources, and edited by the authors. All interpretations, conclusions, and scientific claims are the sole responsibility of the authors. This disclosure is made in accordance with Frontiers in Microbiology editorial policy.

#### 2.6.5 Power considerations

Formal prospective power analysis is not directly applicable to a single-donor co-culture design with *n* = 3 technical replicates per condition, as the biological unit of observation is the condition mean rather than an independent donor. The design was sized to enable comprehensive factorial coverage (10 strains × 2 viability states × 3 concentrations × 3 technical replicates) within a single controlled experiment rather than to achieve a pre-specified statistical power for a primary endpoint. For effects of the magnitude observed, 30 condition means per group are ample; no prospective power calculation was performed, and *post-hoc* power is not reported because it is a deterministic function of the observed effect (310.8-fold TNF-α, 69.4-fold MIP-1β) by orders of magnitude even at the lower bounds of their confidence intervals, 36.4-fold and 2.0-fold respectively. For smaller effects—including strain × dose interactions and FED7 convergence ratios—the three-replicate design provides descriptive precision rather than inferential power, consistent with the exploratory and hypothesis-generating framing of this study. Multi-donor sample size: based on the inter-donor cytokine production variability reported by Li et al. (2016); CV 20%−35% for stimulated PBMC), a minimum of 5–8 biological donors would be required to detect strain × dose interaction effects of 1.5-fold magnitude at 80% power (α = 0.05). This estimate informs the multi-donor validation plan described in Section 4.8.

### 2.7 Machine learning analysis

#### 2.7.1 Classification models

Four algorithms were evaluated for binary classification of bacterial viability status: Logistic Regression with L2 regularization, Random Forest, Extreme Gradient Boosting (XGBoost; Hong and Zhou, 2022 and Chen and Guestrin, 2016), and Support Vector Machine with radial basis function kernel (Cortes and Vapnik, 1995). Hyperparameters for Logistic Regression and Random Forest were optimized by grid search with 5-fold stratified cross-validation using AUC-ROC as the optimization criterion; the Support Vector Machine and XGBoost were run at library default settings, which are declared in the deposited configuration file. This corrects the previous version, which described exhaustive grid search for all four algorithms. Pairwise model comparisons were performed using McNemar's exact test; note that with 60 condition means a difference of one prediction between classifiers is not statistically distinguishable. Feature importance was derived from model coefficients (Logistic Regression), Gini importance (Random Forest, XGBoost), and permutation importance (Support Vector Machine). All stochastic procedures used a fixed random seed (seed = 42) for reproducibility.

#### 2.7.2 Strain classification and functional annotation

Random Forest classification (n_estimators = 500, class_weight = ‘balanced', max_features = ‘sqrt') was additionally applied to: (a) classify strains from viable preparations and compute cytokine feature importance by mean decrease in impurity; and (b) classify functional clusters from hierarchical clustering. All classification performance was evaluated by Leave-One-Out cross-validation (LOO-CV) and compared against the appropriate chance level (10% for 10-class strain, 33.3% for 3-cluster). Feature importance values in Figure 1B are reported as mean ± SD across 30 independent model fits generated by varying the random seed [seeds 0–29, i.e., range (30) as declared in the deposited configuration], to characterize the stability of importance rankings across stochastic variation in tree construction. Strain-specific immunomodulatory profiles were characterized using z-score standardization and functional composite scores for five predefined cytokine groups: pro-inflammatory (TNF-α, IL-1β, IL-6, and IFN-γ), anti-inflammatory (IL-10, IL-4), Th1-associated (IFN-γ, IL-2, and IL-12p70), Th2-associated (IL-4, IL-5, and IL-13), and chemoattractant (IL-8, MCP-1, and MIP-1β).

**Figure 1:**
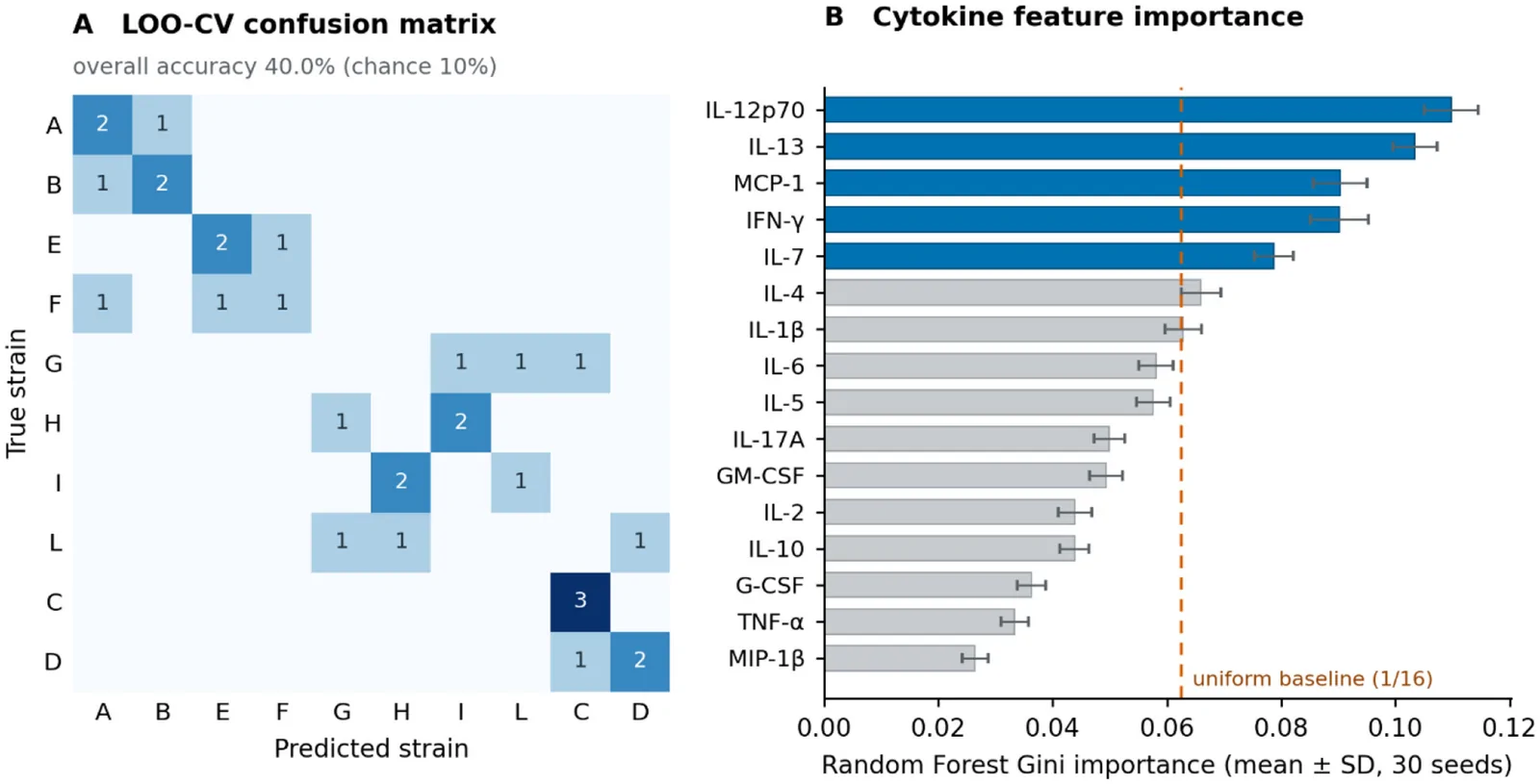
Random Forest strain classification and feature importance (IL-8 excluded). **(A)** LOO-CV confusion matrix for 10-strain classification of viable preparations (30 conditions; chance level 10%). Classification performed on 16-cytokine profiles (IL-8 excluded due to upper-quantification-limit saturation in the majority of viable conditions). Overall LOO-CV accuracy: 40.0% (95% CI 24.6–57.7%; chance 10%). Rows: true strain; columns: predicted strain. **(B)** Cytokine feature importance (mean decrease in impurity ± SD across 30 independent model fits). Top discriminators: IL-12p70 (0.110), IL-13 (0.103), MCP-1 (0.090), IFN-γ (0.090), IL-7 (0.079). Dashed red line: uniform baseline (1/16). Top discriminators differ from the highest absolute fold-change cytokines (IL-6, IL-1β, G-CSF), indicating that strain identity information resides in immune polarization direction, not activation magnitude.

The derived quantities used throughout are defined as follows. Functional Equivalence Dose: for strain s and analyte c, let FC(s, v, d, c) be the fold-change relative to the unstimulated control for viability state v at concentration d; FED7(s, c) is the lowest heat-treated concentration d, scanned in ascending order over 105−107 TFU/well, satisfying FC(s, HT, d, c) ≥ 0.70 × FC(s, viable, 106, c). The criterion is applied per analyte rather than to a composite score, is evaluated over the 14-analyte subset, and uses 106 AFU/well as the viable reference; cells are reported as not applicable where the viable reference is not positive, and as not reached where no tested concentration meets the criterion. Monotonicity percentage: the proportion of strain × analyte combinations, over all 17 analytes, for which the three condition means increase strictly with concentration, FC(105) < FC(106) < FC(107), computed separately within each viability state. Convergence ratio: the maximum heat-treated fold-change across the three concentrations divided by the viable fold-change at the 106 AFU/well reference, expressed as a percentage. Composite scores are the mean of the z-standardized fold-changes of their member analytes, and the Th1/Th2 ratio is the quotient of the Th1 and Th2 composites. The composite scores reported here use the overlapping definitions given above, in which IFN-γ contributes to both the pro-inflammatory and Th1 scores and IL-4 to both the anti-inflammatory and Th2 scores, reflecting their dual functional roles; these definitions are authoritative and the deposited configuration file will be aligned to them.

#### 2.7.3 Bayesian estimation of immunostimulatory probability

The probability of each experimental condition producing immunostimulatory responses was estimated using a Bayesian framework with Jeffrey's non-informative prior [Beta (0.5, 0.5)]. For each strain × viability × concentration combination, a cytokine was classified as “elevated” if its mean concentration exceeded 150% of the negative control median. The 150% threshold was selected as a conservative criterion representing a 1.5-fold elevation above baseline, consistent with the minimum biologically meaningful fold-change in PBMC immunoassay systems given typical intra-assay variability of 15%−20%. Jeffrey's non-informative prior [Beta (0.5, 0.5)] was selected to minimize prior influence on posterior estimates; sensitivity analyses using a uniform prior [Beta (1, 1)] and a more conservative threshold (200% of control median) yielded qualitatively equivalent results, and preserved the relative ordering of conditions. As expected, a stricter threshold lowers the absolute posterior values; the numerical floor stated in the previous version was inconsistent with the primary analysis (minimum 0.64) and has been withdrawn. The number of elevated cytokines (out of 17) was used as the success count in a binomial model, yielding posterior Beta distributions for the probability of immunostimulation. It is acknowledged that the binomial model assumes independence between cytokine responses, an assumption that is not fully met given the high inter-cytokine correlations observed in this dataset (Spearman ρ up to 0.97; Section 3.8). The resulting posterior distributions should therefore be interpreted as descriptive summaries of response breadth rather than as independent probabilistic confirmation of the frequentist findings.

## 3 Results

### 3.1 Data quality and distribution characteristics

The genome-wide immunomodulatory overview is shown in Figure 2A (heatmap) and Figure 2B (pathway-level radar). Data quality metrics, out-of-range handling and imputation, and distributional characteristics (Shapiro–Wilk normality testing) are reported in the Supplementary Material (Section S2); all downstream analyses met the stated quality criteria.

**Figure 2:**
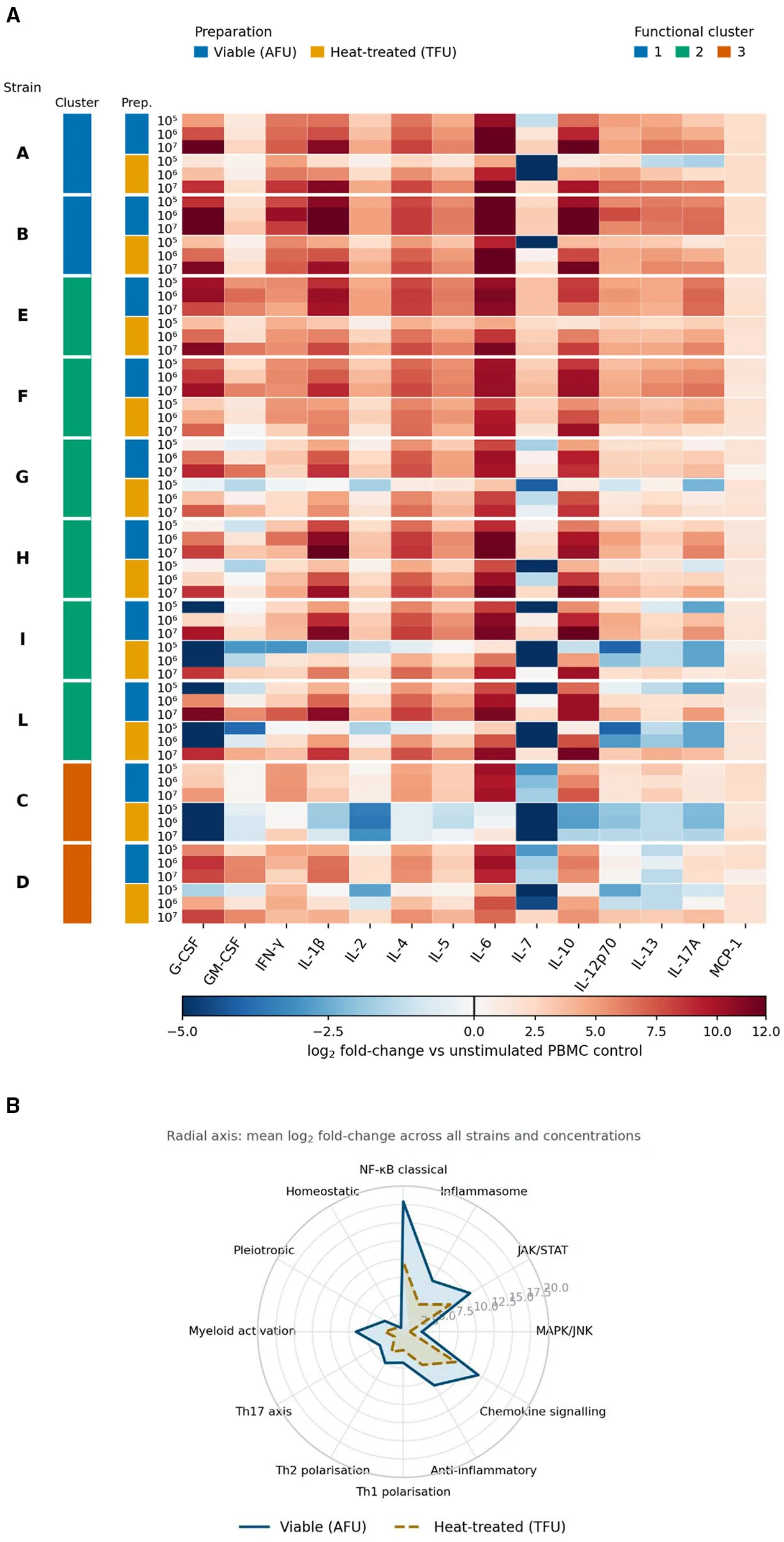
**(A)** Overview of immunomodulatory activity across all strains, doses and preparation types. Heatmap of mean log_2_ fold-change (*n* = 3 technical replicates) for 14 quantifiable cytokines relative to the unstimulated PBMC control (IL-8, MIP-1β, TNF-α excluded; see Supplementary Figure 1). Rows: strain (A–L), dose (10^5^, 10^6^, 10^7^ cells/well), preparation type (V = viable/AFU; HT = heat-treated/TFU). Color: diverging RdYlBu, capped at log_2_ = −5 (lower) and +12 (upper). White lines delineate strains. **(B)** Pathway-level radar visualization of the same dataset. Mean immunomodulatory activity is summarized across twelve functional pathway categories for viable **(left)** and heat-treated **(right)** preparations, averaged across all ten strains at each of the three dose levels (10^5^ green solid, 10^6^ orange dashed, 10^7^ red dash-dotted). Each spoke represents one functional grouping of the 17-plex BioPlex panel: MAPK/JNK, JAK/STAT, inflammasome, NF-κB classical, homeostatic, pleiotropic, myeloid activation, Th17 axis, Th2 polarization, Th1 polarization, anti-inflammatory, and chemokine signaling. Independent radial scales are used for the two panels to accommodate the higher absolute activation magnitude of viable preparations. The concentric expansion of the polygons with increasing dose visualizes the monotonic dose-dependent intensification of pathway engagement common to both preparation types. Anti-inflammatory, chemokine and inflammasome modules emerge as the dominant axes of response, while homeostatic and Th1-polarizing modules show comparatively limited engagement. Pathway-category groupings represent a functional aggregation for visual integration; quantitative pathway-level inference is not derived from these aggregations.

### 3.2 Live bacteria induce profoundly greater cytokine responses than heat-treated preparations

TNF-α showed the largest fold-change (310.8-fold higher in viable conditions; 95% CI 36.4–2,653), followed by MIP-1β (69.4-fold; 2.0–2,435), IL-8 (25.8-fold; 3.3–205), G-CSF (15.5-fold; 5.8–41.4), IL-1β (13.3-fold; 8.6–20.7), IL-10 (9.5-fold; 4.5–19.8), IL-6 (8.9-fold; 4.2–18.9), IL-7 (6.1-fold; 2.9–12.8) and IL-4 (5.3-fold; 3.2–8.7; Supplementary Figure 2). Values are geometric-mean ratios on the 30 condition means with 95% confidence intervals. The three widest intervals in the panel belong to TNF-α, MIP-1β and IL-8, precisely the three analytes that exceeded the upper limit of quantification in a substantial proportion of viable conditions; the direction and order of magnitude of those three effects are secure, their exact multiples are not, and the intervals quantify what the imputation costs. Pro-inflammatory innate cytokines were disproportionately affected: TNF-α, IL-1β, and IL-6 collectively demonstrated fold-changes exceeding 9-fold, a pattern that would be consistent with NF-κB-dependent transcriptional activation by metabolically active bacteria, although no transcriptional or pathway readout was collected in this study. Among adaptive cytokines, IFN-γ (3.0-fold), IL-2 (3.8-fold), and IL-12p70 (3.8-fold) were significantly elevated in viable conditions, indicating Th1 activation. IL-4 (5.3-fold), IL-5 (3.1-fold), and IL-13 (2.4-fold) were also significantly higher, suggesting parallel Th2 activation rather than selective polarization. IL-17A (5.1-fold) was also significantly elevated in viable conditions. Under the paired analysis both IL-7 and IL-13 reach significance (*q* = 0.0001 and *q* = 0.0013 respectively); Both were also significant under the unpaired test used in the previous version; pairing sharpens the estimate rather than changing the direction of the conclusion, and 16 of 17 analytes differ under either test. MCP-1 (CCL2) was the sole analyte showing no significant difference between viability states under the paired analysis (Wilcoxon *q* = 0.98; geometric-mean ratio 0.99, 95% CI 0.89–1.12, a bounded null that excludes any difference larger than approximately 11% in either direction). All *p*-values reported in this section are descriptive indicators within a single-donor experiment and should not be interpreted as inferential estimates of population-level effects. The interpretation of this exception is developed in Section 4.6. MCP-1 is a monocyte chemoattractant produced through both MyD88-dependent and MyD88-independent signaling pathways, and its constitutive-like expression in both conditions suggests that the mere physical presence of bacterial structural components—regardless of viability—provides a sufficient stimulus for MCP-1 secretion. The viability-independence of MCP-1 across all ten strains and three concentrations constitutes an internal methodological control, consistent with the differential responses observed for the other 16 analytes reflecting biological discrimination rather than experimental confounding, although a single invariant analyte cannot formally exclude confounding (Section 4.6).

All 16 significantly different cytokines exceeded both the statistical threshold (FDR < 0.05) and the magnitude threshold (absolute log_2_ fold-change > 0.585, i.e., a 1.5-fold difference), indicating biologically meaningful as well as statistically significant effects. All 16 significant analytes were elevated in viable conditions at the panel level; at the level of individual strain x dose combinations, however, heat-treated preparations exceeded viable preparations in three instances (Section 3.7). The direction of the panel-level effect is therefore consistent with generalized amplification mechanism rather than qualitative immune reprogramming.

#### 3.2.1 Bayesian estimation of response breadth by viability state

Bayesian estimation provided a complementary description of response breadth: live bacteria conditions achieved P(immunostimulatory) ranging from 0.64 (Strain L at 105 AFU/well) to 0.97, with 16 of 30 conditions reaching the maximum posterior probability of 0.97 (17/17 cytokines elevated above 150% of control median). In contrast, heat-treated conditions ranged from *P* = 0.19 (Strains C and I at 105 TFU/well) to *P* = 0.97 (Strains E and F already at 105 TFU/well; Strains B, D, H, L at 107 TFU/well), demonstrating that the immunostimulatory gap between viability states is dose-dependent and strain-modulated (Figures 3, 4).

**Figure 3:**
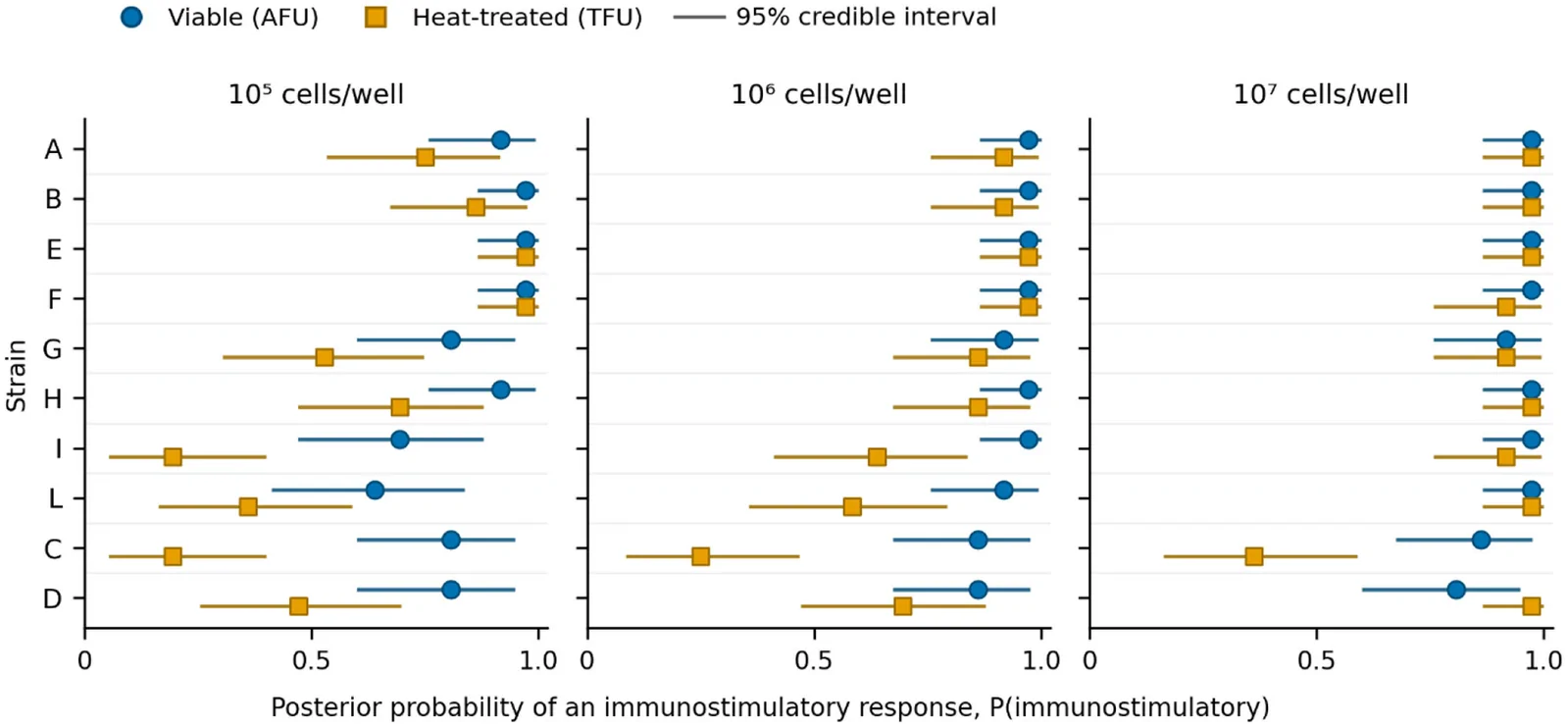
Posterior probability of an immunostimulatory response by strain, preparation and concentration. Points show the posterior mean of P(immunostimulatory) under a Beta-binomial model with a Jeffreys prior; horizontal bars show the 95% credible interval. Circles (blue): viable preparations (AFU); squares (orange): heat-treated preparations (TFU). Panels, left to right: 105, 106 and 107 cells/well. Strains are ordered as in Figure 10A. Per-condition counts of elevated analytes are given in Figure 4.

**Figure 4:**
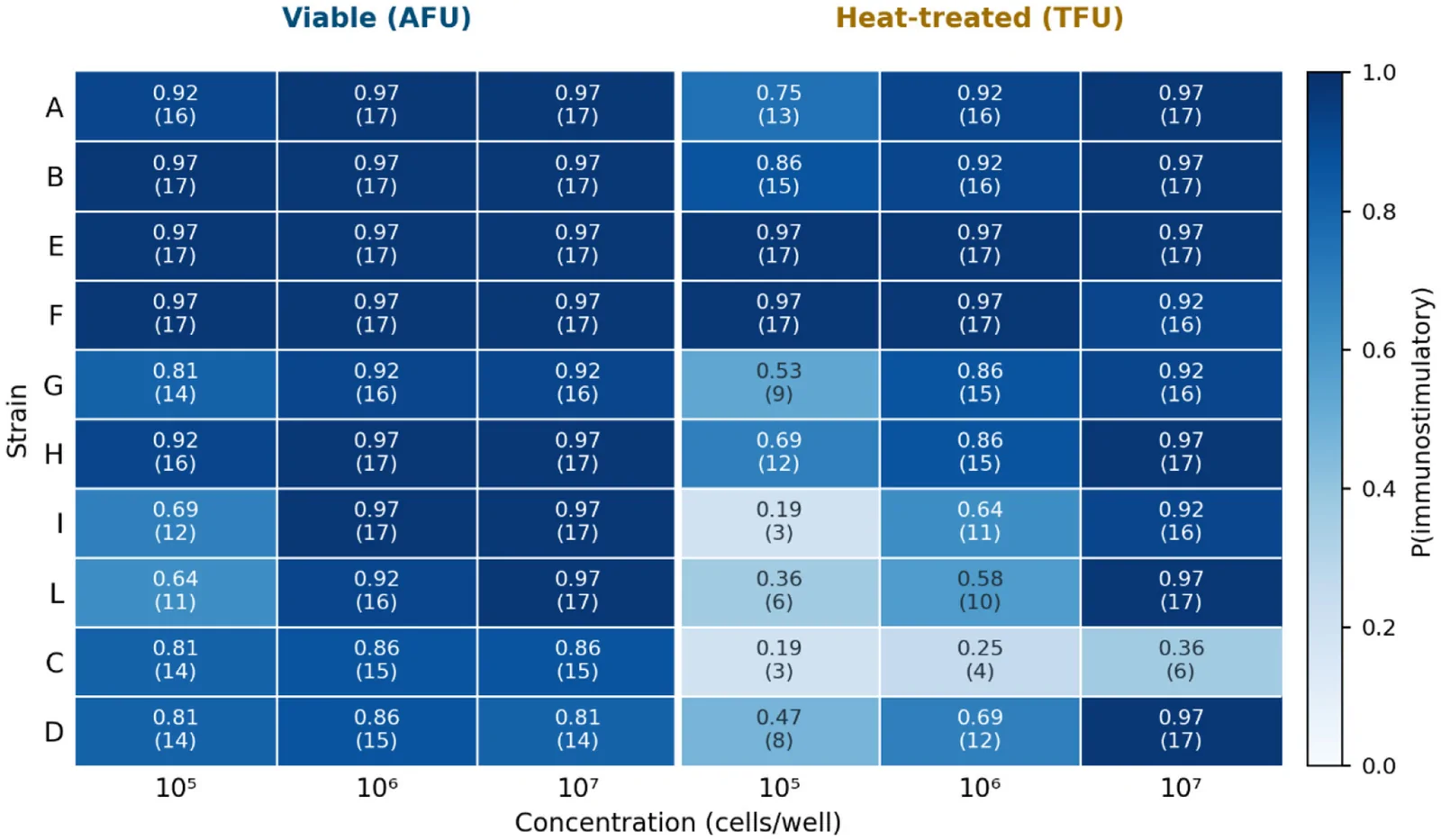
Posterior probability of an immunostimulatory response, strain × concentration matrix. Each cell gives the posterior mean of P(immunostimulatory) for one strain at one concentration, with the number of analytes exceeding 150% of the control median (of 17) in parentheses. Left block: viable preparations (AFU); right block: heat-treated preparations (TFU). Columns within each block: 105, 106 and 107 cells/well. Color scale: sequential single-hue blue, 0–1. The same posteriors are shown with their credible intervals in Figure 3.

### 3.3 Dose-response relationships differ qualitatively between viability states

The second major finding—addressing whether measurable dose-response relationships exist—revealed an unexpected and methodologically significant asymmetry. Heat-treated preparations demonstrated stronger dose-response correlations overall: the strongest mean within-strain Spearman correlations were observed for IL-4 (mean ρ = 1.00), MIP-1β (mean ρ = 0.97), G-CSF (mean ρ = 0.97), IL-1β (mean ρ = 0.95), IL-10 (mean ρ = 0.95), and IL-6 (mean ρ = 0.95) in the heat-treated conditions. Given that Spearman rank correlation with three dose levels can only assume discrete values (0, ±0.5, and ±1.0), formal *p*-value testing is not appropriate; direction and magnitude are reported descriptively. Heat-treated preparations showed stronger dose-response correlations than viable preparations for 13 of 17 cytokines when assessed as mean within-strain Spearman rank correlations across the three dose levels. Viable preparations, despite inducing substantially greater absolute cytokine levels at every concentration, exhibited weaker dose-response correlations across most analytes.

We hypothesize that live bacteria proliferate during incubation: even the lowest inoculum (105 AFU/well) may have expanded substantially during the experiment, compressing the effective difference between the three nominal dose levels. This hypothesis is consistent with the observed asymmetry but was not directly tested in this study, as endpoint bacterial enumeration was not performed. The stronger dose-response correlations observed for heat-treated preparations are not evidence that heat-treated bacteria are intrinsically more dose-responsive—they are evidence that the input variable is accurately controlled only for heat-treated preparations. Kruskal-Wallis tests indicated overall concentration effects for 14 of 17 cytokines (all except IFN-γ, IL-12p70, and MCP-1) in heat-treated conditions when tested on condition means (*n* = 10 strains per dose level); cytokines not reaching significance (IFN-γ, IL-12p70, and MCP-1) showed either consistently low absolute responses or high inter-strain variability across dose levels, which is a further description of the same concentration effect summarized by the Spearman analysis rather than an independent confirmation of it. This finding is discussed further in Section 4.

#### 3.3.1 Strain-specific dose-response profiles

Strain-specific dose-response heatmaps revealed marked heterogeneity in dose-sensitivity patterns across the 10 strains (Figure 5). Strains A and B showed monotonically increasing responses (ρ = +1.0) for the majority of cytokines in both viability states, indicating robust dose-dependency regardless of preparation type. In contrast, Strain E showed near-absent dose-response in live conditions (mean ρ = 0.12 across the 16 analytes used for classification), suggesting that its immunomodulatory effect saturates at lower bacterial concentrations. Heat-treated preparations demonstrated stronger dose-response than viable preparations for the majority of strains. Across the panel, 15 of 17 analytes reached significance for the concentration-response correlation in heat-treated preparations against 9 of 17 in viable preparations, and only two heat-treated correlation intervals included zero against five viable—the pattern expected if replication of live bacteria during co-culture erases the inoculum they were given. The magnitude of this difference was strain-dependent, ranging from negligible (Strains A, I, and L) to substantial (Strains B, D, and E).

**Figure 5:**
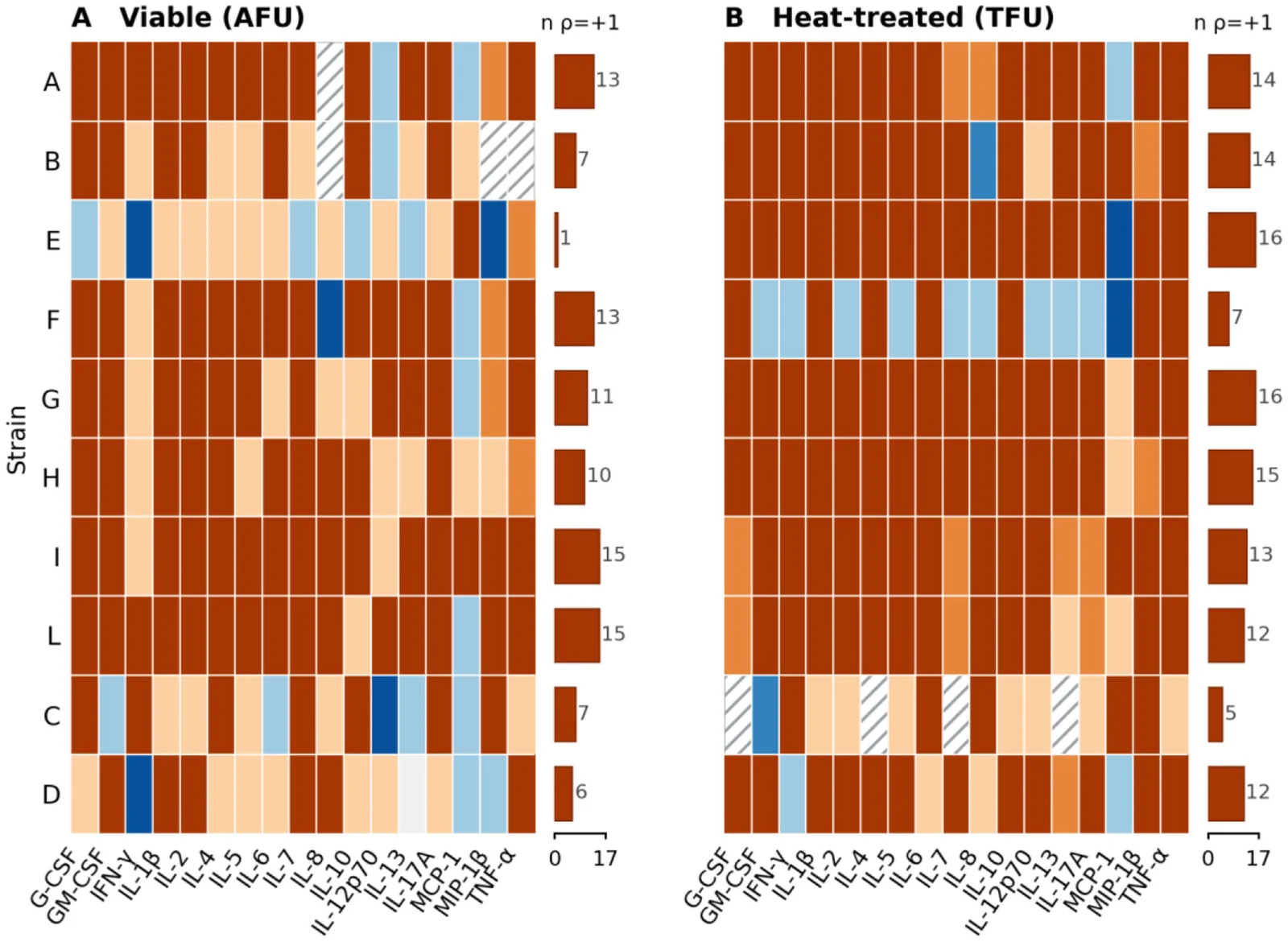
Within-strain dose–response Spearman ρ—categorical representation, all 17 analytes. Spearman rank correlation between bacterial concentration (105, 106, 107 cells/well) and cytokine fold-change for each strain, calculated on condition means (*n* = 3 dose-level means per strain per viability state; all 17 analytes shown). **(A)** Viable preparations. **(B)** Heat-treated preparations. With exactly three ordered dose levels, Spearman ρ is constrained to five discrete values only: −1.0, −0.5, 0.0, +0.5, and +1.0; a categorical color scale is used accordingly. Dark green (ρ = +1.0): monotonically increasing dose-response across all three concentrations. Dark red (ρ = −1.0): monotonically decreasing. Gray (N/A): all values at LLOQ floor (Strain C heat-treated). Across the panel, 58% of strain–cytokine combinations showed monotonically increasing responses in viable preparations vs. 73% in heat-treated preparations.

### 3.4 Dose-response profiles of viable preparations

Per-strain dose-response profiles of viable preparations are summarized in Figure 6, shown in full for all 10 strains in Supplementary Figures 4, 5, and reported in full in the Supplementary Material (Section S3).

**Figure 6:**
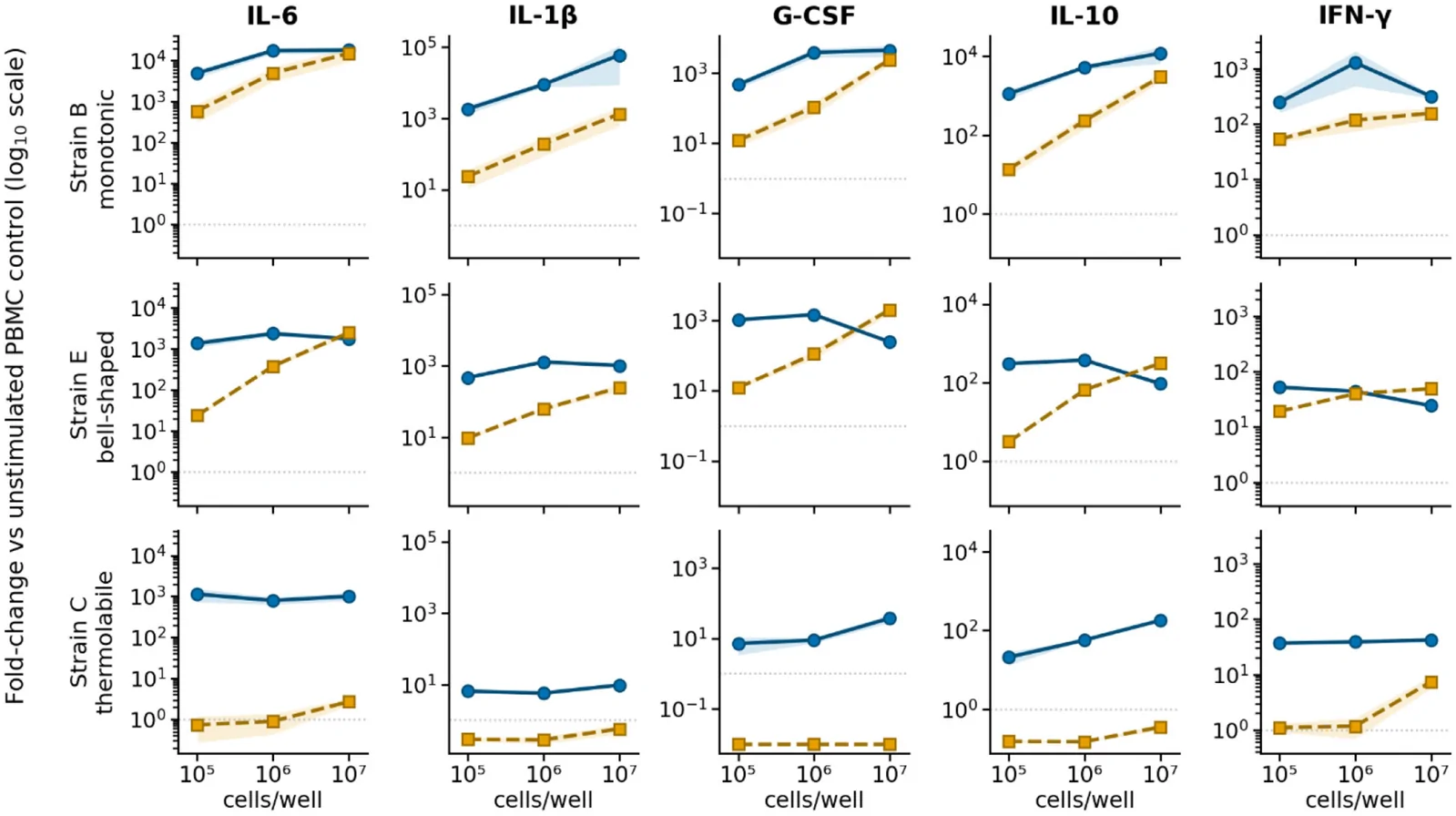
Dose-response profiles of viable and heat-treated preparations across representative strains. Mean fold-change ± SD (*n* = 3 technical replicates) for five cytokines (IL-6, IL-1β, G-CSF, IL-10, IFN-γ) across 10^5^, 10^6^, 10^7^ cells/well. Solid lines with circles: viable preparations (AFU/well). Dashed lines with squares: heat-treated preparations (TFU/well). Y-axis: log scale. Rows: Strain B (strong monotonic), Strain F (moderate monotonic), Strain E (bell-shaped), Strain C (viability-dependent). Dotted horizontal line: control baseline (fold-change = 1). Values represent means of *n* = 3 technical replicates; shaded bands represent ±1 SD.

### 3.5 Non-monotonic dose-response profiles: bell-shaped kinetics

Strains exhibiting non-monotonic, bell-shaped dose-response kinetics are illustrated in Figure 6 and described in detail in the Supplementary Material (Section S4).

### 3.6 Strain C: a strongly viability-dependent immunomodulatory profile

The strongly viability-dependent immunomodulatory profile of Strain C, active in the viable state, with attenuated activity after heat treatment, is detailed in the Supplementary Material (Section S5); this observation is examined further in the Discussion in relation to heat-treated preparations.

### 3.7 Viable vs. heat-treated: dose-dependent convergence and FED7

Systematic comparison of viable (AFU) and heat-treated (TFU) preparations across matched concentration points revealed concentration-dependent convergence of functional activity. At 105 cells/well, HT/V activity ratios for IL-6 ranged from very low (Strain C) to 20% (Strain F). At 10^6^, Strain F HT reached 58% of viable activity. At 10^7^: Strain B HT reached 86% of viable IL-6 (14,980 vs. 17,455-fold at the 106 AFU/well reference); Strain E HT exceeded viable at 10^6^ reference (2,512 vs. 2,383-fold, 105%); Strain H HT reached 103% at matched dose; Strain A HT at 10^7^ TFU produced 266% of viable IL-6 at the 10^6^ AFU reference.

To operationalize this relationship, we introduce the *Functional Equivalence Dose* (FED^70^)—the minimum TFU/well concentration at which a heat-treated preparation achieves ≥70% of the immunological activity of the corresponding viable preparation at the reference dose of 10^6^ AFU/well. The 70% threshold was selected on two grounds: biologically, it represents a conservative threshold acknowledging that PBMC assay variability—both technical and inter-donor—typically ranges from 20 to 35% (Li et al., 2016; van Hemert et al., 2010), making this threshold a pragmatic operational criterion for functional equivalence screening in a single-donor context; regulatorily, it represents a conservative adaptation of the 80%−125% bioequivalence acceptance range established by European Medicines Agency (2010). The FED^70^ matrix (Figure 7) offers a candidate operational basis for postbiotic dose selection. It is proposed as a screening metric with a pre-declared threshold, not as a validated benchmark, and would require multi-donor confirmation before it could inform dose justification. It is noted that for strains exhibiting bell-shaped kinetics (Strains D and E), 106 AFU/well coincides with the peak of the viable dose-response profile. The FED^70^ calculated for these strains therefore represents equivalence to peak viable activity—a more stringent criterion than for monotonically increasing strains, where 106 AFU/well represents an intermediate rather than maximal dose. This asymmetry should be considered when comparing FED^70^ values across strain phenotype groups. Specifically, FED^70^ values for Strains D and E represent equivalence relative to peak viable activity, whereas FED^70^ values for monotonically increasing strains (A, B, F, H, I, and L) represent equivalence relative to an intermediate viable dose. These values are therefore not directly comparable across phenotype groups and should be interpreted within each strain's dose-response context individually.

**Figure 7:**
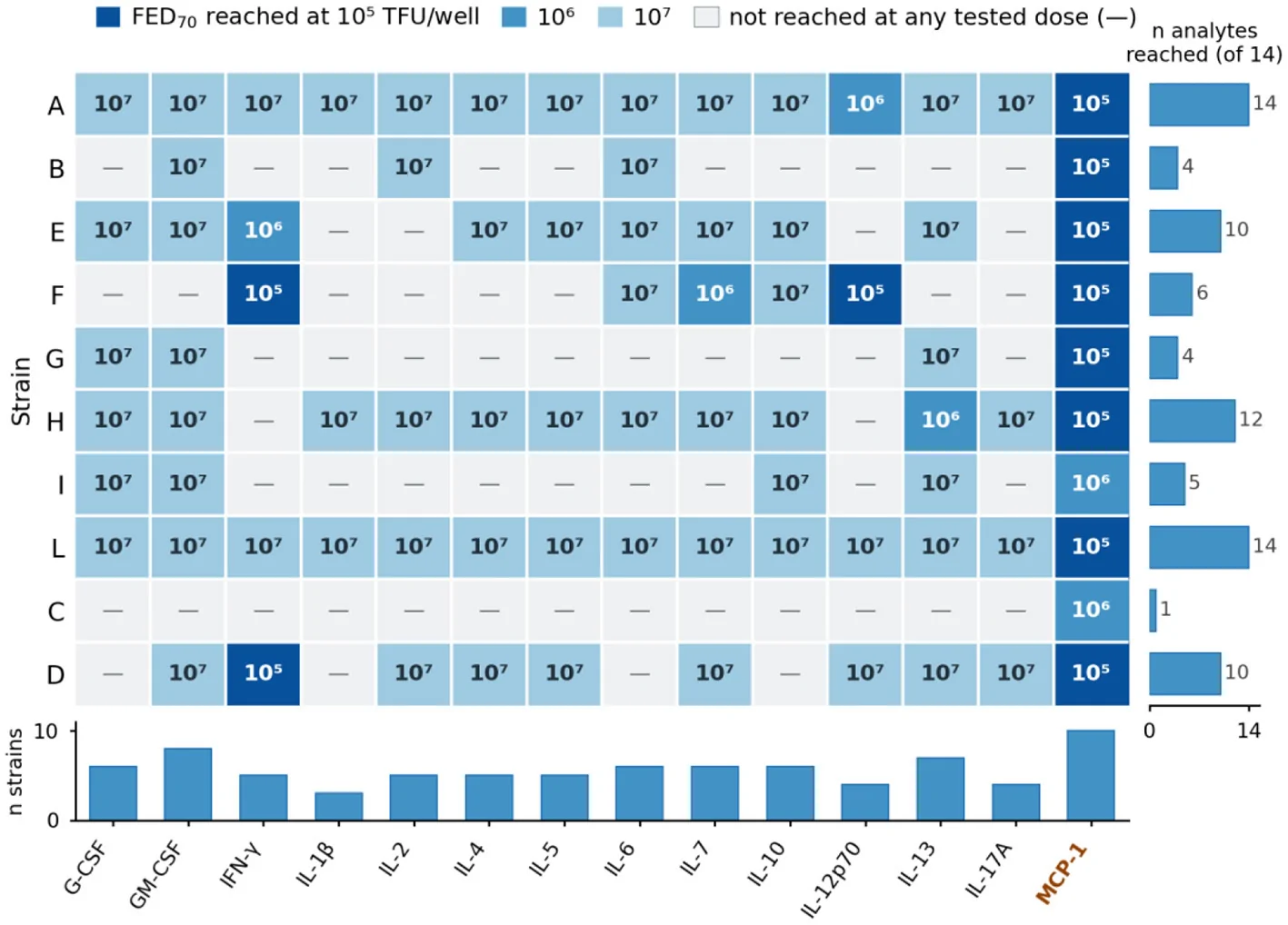
Functional equivalence dose (FED^70^) matrix. Minimum TFU/well concentration at which heat-treated preparations achieve ≥70% of the immunological activity of the corresponding viable preparation at the reference dose of 106 AFU/well, for each strain (rows A–L) and 14 quantifiable cytokines (columns; IL-8, MIP-1β, TNF-α excluded). Color coding: gray = N/A (viable response < 1.5 × control, equivalence undefined); red = “Not reached” (viable response sufficient but HT did not achieve ≥70% equivalence at any of the three tested doses); light green = FED7 at 105 TFU/well; teal = FED^70^ at 10^6^ TFU/well; dark blue = FED^70^ at 10^7^ TFU/well. Data represent means of *n* = 3 technical replicates.

### 3.8 Inter-cytokine correlation structure is dominated by global response amplitude

Inter-cytokine Spearman correlation analysis across the full dataset revealed highly structured co-regulation patterns reflecting coordinated immune response programs (Figure 8). Among the strongest correlations were the Th2/adaptive pairs IL-2 and IL-5 (ρ = 0.97), IL-4 and IL-5 (ρ = 0.95), and IL-2 and IL-4 (ρ = 0.94). These near-unity correlations across the entire dataset reflect global response amplitude as the dominant source of co-variation: conditions of high bacterial stimulation (viable preparations at high dose) produce uniformly elevated responses across the cytokine panel, while low-stimulation conditions produce uniformly reduced responses. This dataset-level correlation is therefore an ecological phenomenon driven by the viability and dose factors rather than evidence of direct transcriptional co-regulation between individual cytokine pairs. Within-condition correlations, stratified by viability state and dose level, are expected to be substantially lower and would more directly reflect pathway-specific co-regulation.

**Figure 8:**
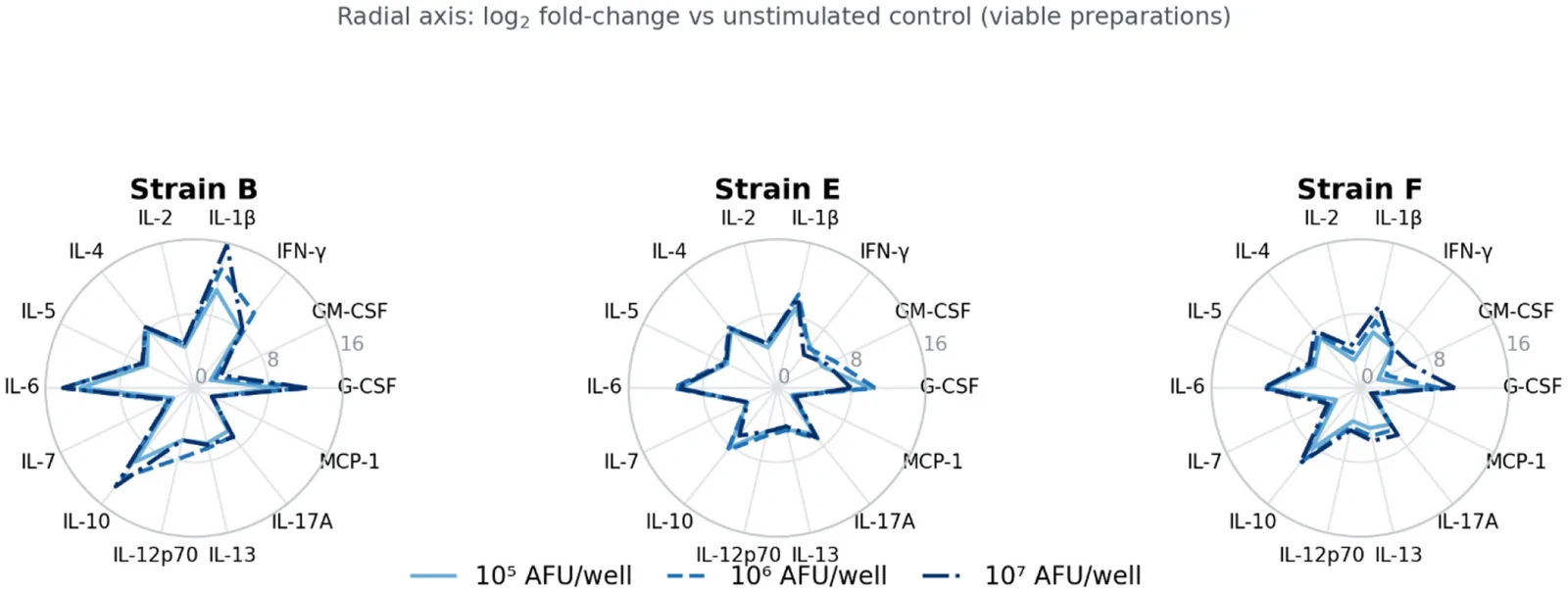
Dose-dependent cytokine co-regulation profiles (viable preparations). Radar charts for Strains B, E, and F across viable dose levels (10^5^, 10^6^, and 10^7^ AFU/well). Spokes represent individual cytokines (14 quantifiable analytes); radial distance represents log_2_ fold-change versus unstimulated control, clipped at zero (negative values not shown). Concentric rings correspond to log_2_ fold-change values of 3, 6, and 9 (i.e., linear fold-changes of 8 ×, 64 ×, and 512 × vs. unstimulated control). Polygons: light shading = 105 AFU/well; medium shading = 10^6^ AFU/well; dark teal = 10^7^ AFU/well. Values represent means of *n* = 3 technical replicates. Strain B illustrates non-isometric dose-dependent expansion with IL-1β and IL-10 scaling more steeply than IFN-γ; Strain E illustrates bell-shaped kinetics with collapse of G-CSF at 10^7^; Strain F illustrates moderate monotonic escalation.

The composition of the two correlation clusters is given in the Supplementary Material (Section S10).

### 3.9 Strain-specific immunomodulatory signatures emerge from data-driven analysis

To characterize the immunological fingerprint space at the strain level, PCA was performed on the matrix of condition means (60 conditions: 10 strains × 2 preparation types × 3 doses), collapsing technical replicates to their mean—a distinct analysis from the full-dataset PCA reported in Section 3.10 which preserves replicate-level variance structure.

PCA of the condition-level matrix (60 conditions: 10 strains × 2 preparation types × 3 doses) revealed that the first two principal components explained 85.2% of total variance (PC1: 75.5%, PC2: 9.7%), demonstrating that the 14-cytokine immunological space is highly structured (Figure 9A). Unsupervised hierarchical clustering of strain-level mean fingerprints (Ward linkage, Euclidean distance) identified three functionally distinct clusters (Figure 9B): Cluster 1—Strains A, B: high-magnitude, broad-spectrum pro-inflammatory cytokine induction; Cluster 2—Strains E, F, G, H, I, L: moderate responses with diverse kinetic profiles; Cluster 3—Strains C, D: low-magnitude viable responses; of these, Strain C alone showed strongly attenuated activity in the heat-treated form (Section 3.6). These cluster assignments reflect the immunological profile of a single donor and should be interpreted as hypothesis-generating phenotypic groupings requiring validation across independent donors.

**Figure 9:**
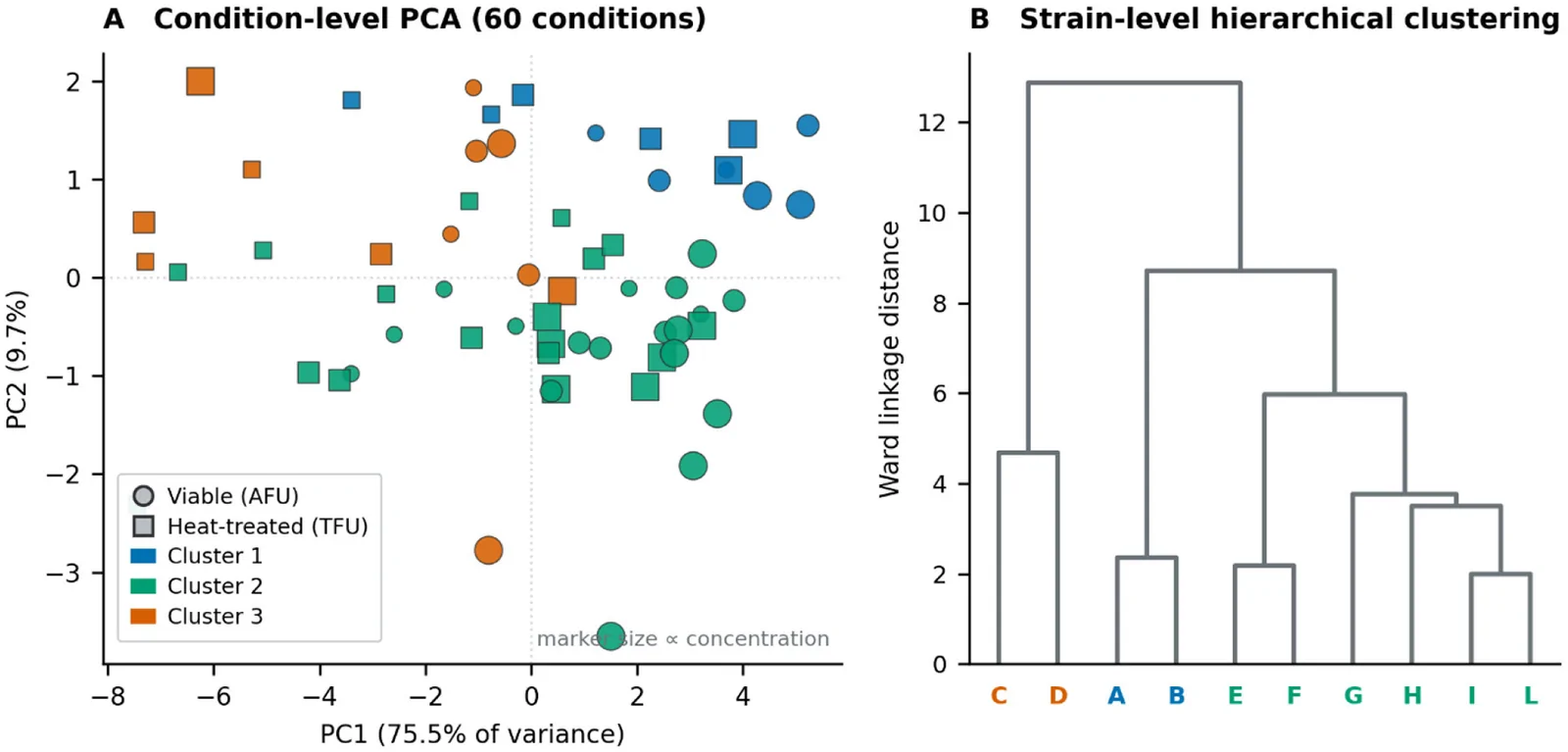
PCA and hierarchical clustering of probiotic immunological fingerprints. **(A)** PCA of all 60 conditions (10 strains × 2 preparation types × 3 doses). PC1: 75.5% variance; PC2: 9.7% (total 85.2%). Circles: viable (AFU); squares: heat-treated (TFU). Marker size proportional to dose. Strain labels within markers; colors by strain. **(B)** Hierarchical dendrogram (Ward linkage, Euclidean distance) of strain-level fingerprints across all conditions (17 analytes). Three clusters: Cluster 1 (high-magnitude: A, B); Cluster 2 (moderate: E, F, G, H, I, L); Cluster 3 (low-magnitude: C, D). Leaf label colors correspond to cluster assignment.

The per-strain composition of each functional group, and the range of inter-strain variation for individual analytes, are reported in the Supplementary Material (Section S11).

Random Forest classification of 10 strains from viable preparations (30 conditions, LOO-CV) achieved 40.0% accuracy (95% CI 24.6–57.7%) vs. 10% chance—approximately a four fold improvement; the 95% confidence interval on the accuracy, 24.6%−57.7%, is consistent with anything between roughly two- and sixfold (Figure 1A). Feature importance analysis (Figure 1B) identified IL-12p70 (importance = 0.110), IL-13 (0.103), MCP-1 (0.090), IFN-γ (0.090), and IL-7 (0.079) as the cytokines carrying the greatest discriminative information for strain classification. Notably, IL-13 ranked as the second strain discriminator despite carrying one of the smallest viability effects in the panel (Section 3.2). IL-13 shows the second-smallest viability effect of any analyte (geometric-mean ratio 2.4, 95% CI 1.4–4.1, above only MCP-1 at 0.99) yet ranks second among strain discriminators. This dissociation reflects the distinction between two different classification questions: IL-13 does not distinguish viability states globally, but carries strain-specific information—levels characteristic of each strain are preserved regardless of preparation type. An analyte can be viability-insensitive yet strain-informative, and this dissociation is precisely what multiplex ML analysis is designed to detect. This ranking diverges from the analytes with the highest absolute fold-changes (IL-6, IL-1β, and G-CSF) and is consistent with the IL-10/IL-12 polarization framework validated by Foligne et al. (2007) and O'Mahony et al. (2005): the cytokines that distinguish strains are those reflecting immune polarization direction, not overall activation magnitude.

Functional cluster classification (3-class, chance 33.3%) achieved 83.3% leave-one-condition-out partition-recovery consistency (95% CI 66.4–92.7%). Because the held-out condition's strain remains represented in training through its other two doses, this figure measures recovery of an internally derived partition rather than generalization. Under leave-one-strain-out cross-validation, in which the held-out strain is absent from training entirely, accuracy was 66.7% against a chance level of 33.3%; this is the appropriate leakage-free estimate of generalization to a strain not used to define the clusters (Figure 10B). Binary preparation-type classification (viable vs. heat-treated, chance 50%) achieved 63.3% LOO accuracy (95% CI 50.7–74.4%), a lower bound only marginally above the 50% chance level, consistent with but not establishing that thermal treatment produces a detectable shift in cytokine-induction signature across the strain panel—implying that a preparation's immunological identity is determined jointly by its strain origin and its viability state. The classification accuracies, together with per-strain F1 scores and the descriptive dose-stratified principal component structure, are consolidated in Figure 11 (panels A and B). It should be noted that these three clusters were derived by unsupervised hierarchical clustering of the same cytokine data used to train and evaluate this classifier. The 83.3% figure therefore represents an internal consistency metric—confirming that Random Forest can recover the hierarchical partition—rather than an independent validation of the clusters as biological phenotypes. External validation on strains not used to derive the cluster definitions would be required to establish generalisability.

**Figure 10:**
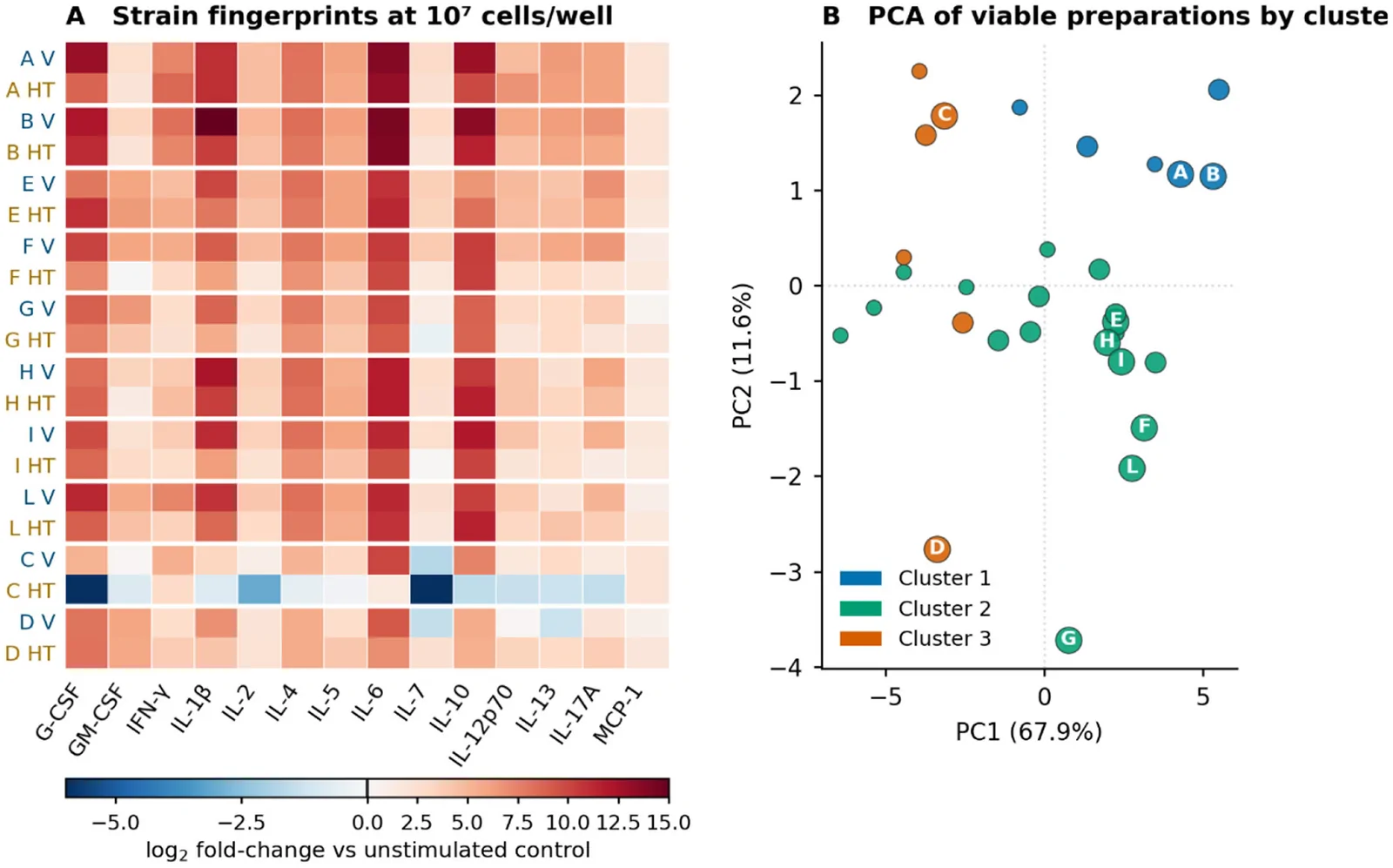
Immunological fingerprint heatmap and cluster validation. **(A)** Heatmap of strain immunological fingerprints at 10^7^ cells/well, ordered by cluster. Rows: viable (V, blue) and heat-treated (HT, orange) for each strain. Columns: 14 cytokines. Color: log_2_ fold-change (RdYlBu). Heavy lines: cluster boundaries. Cluster 1 (high-magnitude: A, B); Cluster 2 (moderate: E, F, G, H, I, L); Cluster 3 (low-magnitude: C, D). **(B)** PCA of viable preparations colored by cluster (Cluster 1: teal; Cluster 2: sage; Cluster 3: red). Marker size proportional to dose. Cluster separation illustrates the partition obtained from these data; biological coherence is not established by this analysis. Partition-recovery consistency: 83.3% (95% CI 66.4–92.7%, leave-one-condition-out); generalization accuracy under leave-one-strain-out cross-validation: 66.7% (95% CI 48.8–80.8%); chance 33.3%.

**Figure 11:**
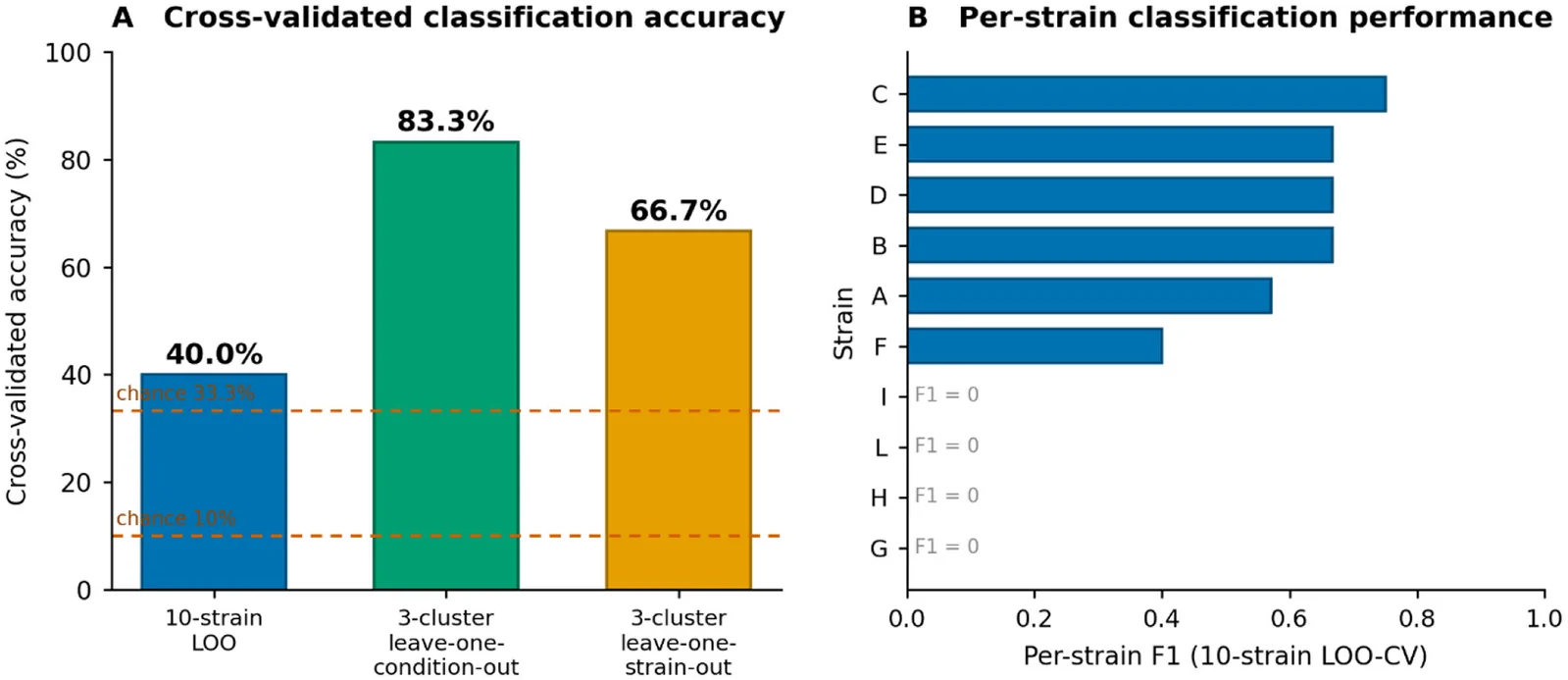
Random Forest classification performance and dose-stratified immunological structure (IL-8 excluded). **(A)** Leave-one-out cross-validated accuracy for 10-strain classification (chance 10%) and for 3-cluster classification (chance 33.3%), computed on condition means (*n* = 60), with per-strain F1 scores. The per-dose accuracy panel presented in the previous version has been withdrawn: within a single dose level each strain contributes exactly one condition, so leave-one-out cross-validation applied at replicate level retains two sibling technical replicates of the held-out sample in the training set. Re-analysis under a leakage-free split, in which the entire condition is held out, reduces per-dose accuracy from 96.7%−100% to 0% (95% CI 0–11.4%), a result indistinguishable from the 10% chance level and confirming that the design does not support strain classification within a single dose level. **(B)** Dose-stratified principal component analysis of viable preparations. Three independent PCAs were performed on the 10 viable strain fingerprints at each dose level (10^5^, 10^6^, 10^7^ cells/well), each panel reporting its own PC1/PC2 explained variance. Markers are colored by functional cluster: teal = Cluster 1 (A, B); sage = Cluster 2 (E, F, G, H, I, L); red = Cluster 3 (C, D). **(B)** is descriptive and involves no cross-validation.

### 3.10 Analyses relocated to the supplementary material

Unsupervised k-means partitioning of the full dataset and a comparison of four classification algorithms for viability status were also performed. Both are descriptive analyses that do not bear on the study's primary questions, and both are reported in full in the Supplementary material (Sections S6 and S7) rather than here. The Bayesian discussion previously in Section 4.1.1 has likewise been relocated, to Supplementary Section S8.

The replicate-level variance decomposition is reported in the Supplementary material (Section S12).

## 4 Discussion

This study was designed to address four interconnected methodological questions that, despite their foundational importance, remain inadequately answered in the probiotic literature: (1) are there measurable and reproducible differences in immunomodulation between live and heat-treated bacteria? (2) can dose-response relationships be demonstrated across a range of concentrations? (3) how does the method of bacterial quantification affect the reliability of dose-response data? and (4) can machine learning tools extract biologically meaningful information from a well-structured factorial experimental design? In this single-donor model the data are consistent with an affirmative answer to each of the four questions, subject to the caveats set out below, while simultaneously exposing the methodological fragility of conventional approaches in the field and raising a fifth question—why the PBMC co-culture model, despite its known limitations, remains the most translationally valid platform for probiotic immunomodulatory screening.

### 4.1 The question of viability: live vs. heat-treated bacteria

The difference in TNF-α secretion between viable and heat-treated conditions is large by any estimator (310.8-fold, 95% CI 36.4–2,653), although its magnitude is imprecisely determined because TNF-α exceeded the upper limit of quantification in a substantial fraction of viable conditions. The direction and the order of magnitude are secure; the exact multiple is not, and we make no claim about how it ranks against previously published comparisons. Rather than simply confirming that live bacteria are “more potent,” this magnitude of difference demands mechanistic interpretation and raises critical questions about experimental design. The 16 of 17 significantly affected cytokines demonstrate that the viability effect is not pathway-specific but represents a global amplification of immune activation. This is consistent with at least four complementary mechanisms unique to living bacteria. First, metabolically active bacteria may continuously release MAMPs through cell division, secretion and autolysis, which would create a sustained and amplifying signal rather than the fixed bolus of ligands presented by a heat-treated preparation; neither MAMP release nor bacterial growth was measured here. Second, live bacteria produce immunologically active metabolites—short-chain fatty acids, lactic acid, hydrogen peroxide, and bacteriocins—that constitute additional stimulatory signals absent from heat-treated preparations (Lebeer et al., 2010). Third, certain PRR pathways may be preferentially activated by viability-dependent structures, such as bacterial messenger RNA recognized by TLR8, which is rapidly degraded in dead cells but continuously synthesized in living bacteria (Akira et al., 2006). Fourth, acidification of culture medium by live bacterial metabolism may modulate immune cell function independently of receptor-mediated signaling.

However—and this constitutes the central interpretive hypothesis of our argument, one that is consistent with but not directly proven by the present data—these same mechanisms that make live bacteria more immunostimulatory also make them uncontrollable as an experimental variable. During a 24-h co-culture at 37 C in nutrient-rich RPMI medium, live bacteria proliferate. The bacteria-to-PBMC ratio at experiment's end is unknown and unknowable without endpoint enumeration, which was not performed in this study and is rarely performed in any published study. A conservative estimate—assuming one bacterial doubling every 60–90 min for the first 6–8 h before nutrient depletion or immune-mediated killing slows growth—suggests that the initial inoculum at 10^7^ AFU/well could have expanded by roughly 16- to 256-fold before reaching stationary phase. This means that comparing “live 10^5^” with “live 10^7^” is not a comparison across a 100-fold concentration range—it may be a comparison across a much narrower effective range once proliferation equalizes the groups. Direct testing of this hypothesis requires endpoint bacterial enumeration—a measurement not performed in this study and rarely performed in any published probiotic co-culture study.

The dose-response evidence bearing on this interpretation, and its practical implication for using heat-treated preparations as a structural-signal control, are developed in the Supplementary Material (Section S13).

### 4.2 The question of dose: systematic dose-response characterization

In this single-donor model the data are consistent with an affirmative answer, subject to critical caveats tied to both quantification methodology and dose-response shape. Across the three tested concentrations (10^5^, 10^6^, and 10^7^ cells/well, corresponding to bacteria-to-PBMC ratios of approximately 0.1:1, 1:1, and 10:1 at the seeding density of 1 x 10^6^ PBMC per well), dose-dependent cytokine induction was demonstrated for the majority of the panel in six of ten strains, extending the limited prior dose-response evidence base (Evrard et al., 2011; Ghadimi et al., 2008; Hua et al., 2010) to the first systematic multi-strain framework. This is itself a novel contribution: Ouwehand (2017) concluded that the probiotic field lacks dose-response evidence for immune endpoints; Shane et al. (2010) criticized the prevalence of single-dose designs; and Christensen et al. (2006), in one of the few multi-dose human probiotic immune studies, found no significant immune changes at any of five dose levels in 71 participants—a null result that our data suggest may reflect CFU-based dose uncertainty rather than genuine dose-independence.

However, the assumption of monotonic dose-response scaling cannot be generalized across all strains. The non-monotonic profiles of Strains D and E—with reductions of 83% (G-CSF) and 25% (IL-6) for Strain E, and of 36 and 50% respectively for Strain D, at 10^7^ vs. 10^6^ AFU/well—are consistent with the hormesis concept proposed by Hua et al. (2010) and the dosage optimum identified by Ghadimi et al. (2008). Several non-mutually exclusive mechanisms could account for this pattern. The most parsimonious candidate involves IL-10-mediated autocrine feedback: substantial IL-10 induction at 106 could create a suppressive milieu reducing IL-6 and G-CSF output at 107 through STAT3/SOCS3-mediated NF-κB suppression (Latvala and Miettinen, 2011). TLR desensitization upon sustained high-density PAMP exposure and induction of negative innate signaling regulators (IRAK-M, A20/TNFAIP3, TOLLIP) represent alternative or complementary candidates (Akira and Takeda, 2004). The present single-timepoint design does not permit mechanistic discrimination between these hypotheses—time-course experiments with IL-10 neutralization or TLR pathway inhibitors would be required to establish causality. The dissociation between viable and heat-treated attenuation patterns at 10^7^ is compatible with several mechanisms, including receptor-level negative regulation, host-cell cytotoxicity at high bacterial density and acidification of the culture medium. Because no host-cell viability, cytotoxicity, cell-recovery or medium-pH measurement was performed, the present design cannot distinguish between them (Section 4.7). A tentative translational implication, which requires multi-donor confirmation before it can inform practice, is that for these strains dose escalation beyond 10^6^ AFU/well did not increase cytokine output and reduced it for several analytes; whether this reflects immunoregulation or reduced host-cell viability cannot be determined here (Section 4.7).

#### 4.2.1 Strain-specific dose-response heterogeneity

The low-concentration response, the regulatory reading of these data and the additional layer of strain-specific dose sensitivity are developed in the Supplementary material (Section S14).

### 4.3 The question of measurement: accurate bacterial quantification

The argument for flow-cytometric enumeration over colony counting, and the regulatory precedent set by the pasteurized *Akkermansia muciniphila* authorization, are set out in the Supplementary material (Section S16).

### 4.4 The question of analysis: machine learning and functional annotation

The rationale for treating the machine-learning layer as a methodological opportunity rather than a limitation is set out in the Supplementary material (Section S11).

The classifier comparison underlying this section is reported in full in the Supplementary material (Section S7).

The functional phenotypes that the machine-learning analysis surfaced, and their relation to the strain-selection literature, are developed in the Supplementary material (Section S11).

### 4.5 Model selection and translational validity

The choice of the PBMC co-culture model as the immunological readout platform for this study was not made by default but reflects a deliberate assessment of translational and analytical requirements that no alternative *in vitro* system can simultaneously satisfy. PBMCs are primary human cells—not immortalized lines carrying oncogenic signaling aberrations. Jurkat T-cell lymphoma cells harbor PTEN and SHIP1 loss resulting in constitutively hyperactivated PI3K/Akt signaling that profoundly distorts T cell receptor-dependent cytokine production (Abraham and Weiss, 2004). THP-1 monocytic leukemia cells exhibit constitutive NF-κB activation, altered TLR1/2/4/6 expression, and quantitatively different cytokine secretion profiles from primary monocytes (Bosshart and Heinzelmann, 2016; Tedesco et al., 2018). These are not minor technical caveats; they are fundamental distortions of the very signaling pathways under investigation.

The heterogeneity of the PBMC population, the reasons the model was chosen despite it, and the extension from a two-cytokine to a seventeen-analyte panel are set out in the Supplementary Material (Section S1).

### 4.6 MCP-1 independence: an internal methodological control

The viability-independence of MCP-1 secretion (Wilcoxon *q* = 0.98; geometric-mean ratio 0.99, 95% CI 0.89–1.12; 13 of 30 pairs in the viable direction) serves as an internal methodological control. Because that interval excludes any difference larger than approximately 11% in either direction, the control rests on a bounded equivalence rather than on a failure to reach significance. MCP-1 (CCL2) is produced through both MyD88-dependent and MyD88-independent signaling pathways and can be activated by bacterial structural components—peptidoglycan, lipoteichoic acid—independently of viable bacterial metabolism. Its consistent secretion across both viability states is consistent with PBMCs having been exposed to comparable structural bacterial material in both conditions. Endotoxin content, host-cell lysis and medium composition were not measured, however, and the invariance of a single analyte cannot formally exclude these as confounders.

The machine-learning reading of MCP-1 independence is given in the Supplementary Material (Section S15).

### 4.7 Limitations

Several limitations warrant transparent acknowledgment. First and most critically, the single-donor design provides no estimate of inter-individual variability and limits generalizability to the broader population. Li et al. (2016) characterized cytokine production variability across 197 donors and identified six genome-wide significant cytokine quantitative trait loci, demonstrating a genetic basis for inter-individual variation. The Milieu Intérieur consortium further established that age, sex, and genetic background are major determinants of cytokine response variation (Patin et al., 2018; Piasecka et al., 2018). Both Foligne et al. (2007) and van Hemert et al. (2010) demonstrated that relative strain rankings are consistent across donors despite absolute value differences, suggesting that the ordinal relationships reported here are likely reproducible, but validation across at minimum five donors of diverse sex, age, and immune backgrounds is necessary before population-level conclusions can be drawn. Specifically, the three functional cluster assignments, the Th1/Th2 polarization ratios, the FED7 thresholds, and the ML classification accuracies reported here all reflect a single immune background. The ordinal relationships between strains are anticipated to be preserved; the absolute quantitative values are not. Multi-donor validation is required before any of these parameters can be used for population-level dose characterization or regulatory substantiation. The ML classification accuracies derive from cross-validation applied to technical replicates within a single-donor experiment. While the dataset size (60–180 observations per classifier) is adequate for model fitting, the structural correlation between technical replicates of the same condition introduces dependency not fully addressed by cross-validation. External validation on independent biological replicates from additional donors is required to confirm generalizability.

Second, the uncontrolled proliferation of live bacteria during the 24-h co-culture represents an inherent limitation of all live bacteria co-culture experiments and should be acknowledged explicitly. Despite flow cytometric enumeration at *T* = 0, the bacteria-to-PBMC ratio at experiment's end is unknown. We have argued that this limitation is paradoxically informative—it provides a plausible mechanistic explanation for why heat-treated preparations show stronger dose-response correlations—but this interpretation remains a hypothesis pending direct experimental verification through endpoint bacterial enumeration.

Third, although the species identity, in-house designation and public culture-collection deposit numbers of each strain are now provided in Supplementary Table 1, whole-genome sequences are not, which precludes genotype-phenotype correlation and identification of the genetic determinants underlying the observed phenotypic differences. Whole-genome characterization will be reported separately. Fourth, substantial out-of-range values (up to 33.9% for MIP-1β above upper limit, 27.8% for IL-7 below detection limit) required imputation, introducing estimation uncertainty particularly at the extremes of the response range; the imputation methods used replaced out-of-range values with the empirically determined quantification boundaries of the assay-specific standard curve, as described in Section 2.6.1. The potential impact of imputed values on multivariate analyses was assessed by verifying that PCA component loadings, cluster assignments, and ML feature importance rankings were qualitatively stable when MIP-1β and IL-7 were excluded from the analysis matrix. The principal multivariate conclusions reported below are not dependent on the imputed values for these two analytes. Sensitivity analyses excluding MIP-1β and IL-7 from PCA, clustering, and ML analyses confirmed that principal component loadings, cluster assignments, and feature importance rankings were qualitatively unchanged. The same sensitivity analysis was also extended to TNF-α and IL-8, the two remaining analytes affected by upper-quantification-limit saturation, since TNF-α carries the study's largest reported fold-change: the principal conclusions were again unchanged, as the principal-component and clustering analyses already use the 14 quantifiable analytes, which exclude these two. Formal quantitative reporting of the remaining sensitivity analyses is deferred to future work with extended concentration ranges that would reduce the proportion of out-of-range values. Fifth, the three-point concentration range precludes formal pharmacological curve fitting (EC50, Hill coefficient); extension to 104 and 108 cells/well would complete the dose-response envelope and enable parametric modeling. Sixth, the 24-h single timepoint captures peak IL-10, IL-12, and IFN-γ production (Kekkonen et al., 2008) but not the temporal dynamics of response initiation or attenuation. Seventh, the PBMC model lacks the epithelial barrier context noted by Kleiveland (2015); the Foligné IL-10/IL-12 correlation was established specifically for Gram-positive bacteria and does not extend to Gram-negative species; *in vivo* validation remains a necessary step. Notably, parallel evaluation of these same strains in an epithelial barrier model revealed fundamentally different response architectures: barrier function was viability-independent (best classification AUC = 0.662) whereas the cytokine responses reported here were viability-dependent (AUC = 0.702); the two AUC values are separated by less than the precision either estimate carries at this sample size, so the contrast between the models is qualitative rather than quantitative, indicating that immunomodulatory and barrier-protective mechanisms have distinct molecular requirements—a dissociation that underscores the necessity of multi-endpoint characterization and will be reported in detail elsewhere. Eighth, the cross-validation approach for machine learning, while appropriate for this dataset size, provides an optimistic estimate of generalization performance compared to validation on an independent external dataset; the ML framework requires validation on independent PBMC datasets per the benchmarking standards of Li and Li (2025) and Zhou and Gallins (2019). Ninth, no positive control stimulus—such as lipopolysaccharide for monocyte TLR4 activation or phytohemagglutinin for T-cell activation—was included in the experimental design. The absence of a positive control means that inter-run PBMC functional competence cannot be formally verified from the reported data. While the cytokine responses observed across the majority of viable-bacteria conditions are consistent with functional PBMC responsiveness, conditions showing low responses—particularly heat-treated Strain C and Strain I at 105 TFU/well—cannot be distinguished with certainty from a globally suppressed PBMC preparation on that experimental day. Future studies employing this framework should include at minimum one positive control stimulus per experimental run.

### 4.8 Toward an evidence-based dose characterization framework

Three operational principles emerge from the totality of findings. First, multi-concentration testing is necessary to characterize dose-response shape: monotonic scaling cannot be assumed without experimental verification, and bell-shaped kinetics are a mechanistically plausible outcome, observed here in two strains and requiring reproducible outcome for a non-negligible subset of strains. Second, viable and heat-treated preparations require independent characterization at matched concentrations: the Functional Equivalence Dose (FED_70_), grounded in biological and regulatory rationale (European Medicines Agency, 2010), offers a candidate screening metric, expressed in units that CFU-based approaches cannot supply for heat-treated preparations. Third, machine learning-based functional classification of probiotic strains by cytokine-induction phenotype provides a data-driven annotation layer complementing the genomic approaches of Orkkatteri Krishnan et al. (2025) and Sun et al. (2022) and enabling evidence-based strain selection. Together, these principles outline a candidate pre-clinical characterization framework which, if validated across donors, could contribute to the dose-response evidence that EFSA requires (EFSA, 2012) and the ICH E4 mandate—supporting the pre-clinical dose-characterization step that precedes the human intervention studies required for health-claim substantiation.

Future studies should build on this framework in five directions. First, multi-donor validation across at minimum five donors of diverse sex, age, and immune backgrounds to establish population-level robustness of the dose-response profiles, cluster assignments, and ML classification accuracy. Second, whole-genome sequencing of the tested strains to enable genotype-phenotype correlation analysis, extending the Random Forest gene-trait matching approach of Meijerink et al. (2010) to the full multi-strain, multi-concentration dataset generated here. Third, time-course experiments capturing cytokine kinetics at multiple intervals (4, 8, 12, 24, and 48 h) to determine whether bell-shaped dose-response parameters are timepoint-dependent. Fourth, intestinal barrier model integration—incorporating epithelial co-cultures or immune-competent organoid systems alongside PBMC—would provide the physiologically complete assessment that the current model cannot offer, though such platforms remain technically immature for screening applications (Bar-Ephraim et al., 2020; Puschhof et al., 2021). Fifth, and most critically, *in vivo* validation of whether the immunophenotypic classification generated by this framework predicts clinical outcomes is the ultimate test of any preclinical screening tool. Only such validation can establish whether the strain-specific response signatures and FED7 thresholds identified here carry any predictive value for clinical outcomes.

*Tenth, no host-cell readout was collected*. PBMC viability, cytotoxicity (for example lactate dehydrogenase release), apoptosis, post-culture cell recovery and culture-medium pH were not measured at any concentration. Consequently the attenuation of cytokine output observed at 10^7^ AFU/well for Strains D and E cannot be attributed to immunoregulatory feedback in preference to reduced host-cell viability or to acidification of the medium by metabolically active bacteria, and the non-monotonic profiles are therefore reported as an observed dose-response shape rather than as an established regulatory phenomenon. Future applications of this framework should include a host-cell viability or cytotoxicity readout and endpoint pH measurement at every concentration, alongside the positive control stimulus noted above.

## 5 Conclusions

This study describes a computationally reproducible analytical framework, evaluated in a single donor, that addresses four questions fundamental to probiotic and postbiotic research. Are there differences between live and heat-treated bacteria? Yes—large and consistent at the panel level in this donor, although heat-treated preparations exceeded viable preparations for individual strains at matched doses (Section 3.7), with fold-changes spanning from 2.4-fold (IL-13, 95% CI 1.4–4.1) to 310.8-fold (TNF-α, 95% CI 36.4–2,653) across 16 of 17 analytes. Can dose-response relationships be demonstrated? Yes—particularly clearly in heat-treated preparations, where the absence of bacterial replication means the input concentration remains controlled relative to its value at *T* = 0, permitting clearer observation of concentration-dependent immune activation (Spearman ρ up to 1.00), and in viable preparations for 6 of 10 strains across the tested 100-fold range. How should bacterial doses be measured? By flow cytometry per ISO 19344:2015, which provides viability-state-independent enumeration; published comparisons report better precision than plate counting (relative standard deviation 6.70 vs. 12.52%) and this is essential for meaningful dose-response characterization and live vs. heat-treated comparison. Can machine learning enhance the analysis of well-structured experimental data? Yes—not only through above-chance viability classification (RF AUC-ROC 0.702) but through the emergence of three functional immunomodulatory phenotypes not part of the original experimental hypothesis, and through the identification of IL-12p70, IL-13, and IFN-γ, not IL-6 or IL-1β, as the primary discriminators of strain identity. These contributions establish a hypothesis-generating methodological and conceptual framework for evidence-based dose characterization, postbiotic functional equivalence assessment, and data-driven strain annotation, grounded in a single-donor model and requiring multi-donor validation before population-level conclusions can be drawn in probiotic and postbiotic research. The strongly viability-dependent profile of Strain C, active when viable but attenuated after heat treatment, should be interpreted in the context of the absence of a positive control stimulus in this study; while the responses observed in viable-bacteria conditions are consistent with general PBMC responsiveness, formal verification of maximal stimulatory capacity on the experimental day was not performed. All findings derive from a single-donor experimental model and constitute hypothesis-generating evidence requiring multi-donor validation before population-level conclusions can be drawn.

## Data Availability

The datasets and complete analysis code generated and analyzed in this study are openly available in the Zenodo repository, DOI: 10.5281/zenodo.22940914 (https://doi.org/10.5281/zenodo.22940914).
