# Supplementary material for: Beyond the billion: dose-response immunophenotyping and machine learning classification of live vs. heat-treated gram-positive probiotic strains in human peripheral blood mononuclear cells—a single-donor proof-of-concept study
Source: Front Microbiol. 2026 Oct 26;17:1943891. doi: 10.3389/fmicb.2026.1943891 (PMC13745699; doi:10.3389/fmicb.2026.1943891)
Supplement: Supplementary file 1 [file Data_Sheet_1.docx]

**Supplementary Material**

**Section S1. Cell Model: Rationale, Validation, and Analytical Gaps**

The peripheral blood mononuclear cell co-culture model was selected deliberately as the immunological readout platform for this study because it uniquely satisfies both the translational and analytical requirements of the experimental design. PBMCs are primary human cells, not immortalized lines with aberrant signaling, such as THP-1 monocytic leukemia cells that exhibit constitutive NF-κB activation and altered Toll-like receptor expression relative to primary monocytes (Bosshart and Heinzelmann, 2016; Tedesco et al., 2018), or Jurkat T-cell lymphoma cells that harbor PTEN and SHIP1 loss resulting in constitutively hyperactivated PI3K/Akt signaling fundamentally distorting T cell receptor-dependent cytokine production (Abraham and Weiss, 2004). Critically, PBMCs constitute a heterogeneous population comprising monocytes, T lymphocytes, B lymphocytes, natural killer cells, and dendritic cells in physiological proportions (Kleiveland, 2015; Maecker et al., 2012). The 17-plex cytokine fingerprint that forms the basis for machine learning classification in this study emerges from the functional cross-talk among these subpopulations: monocytes and dendritic cells produce TNF-α, IL-6, and IL-12 through Toll-like receptor 2 engagement (Trinchieri, 2003), T helper cells generate IL-4 and IFN-γ, and natural killer cells produce IFN-γ mediated by dendritic cell-derived IL-12 through a positive feedback loop that cannot occur in single-cell-type cultures (Vivier et al., 2011; Iwasaki and Medzhitov, 2015). No single cell line can generate this dimensionality (de Jager et al., 2003; Duffy et al., 2014), and without it, data-driven functional classification would lack the biological signal required for meaningful phenotype discrimination. Intestinal organoids are epithelial structures devoid of immune cells by design (Sato et al., 2009; Clevers, 2016; Bar-Ephraim et al., 2020) and therefore measure barrier function rather than the integrated immune response; immune-competent organoid co-cultures remain technically immature, low-throughput, and unstandardized (Dijkstra et al., 2018; Neal et al., 2018), while organ-on-a-chip systems are fundamentally incompatible with the high-throughput factorial screening format required here (Ingber, 2022).

The PBMC model is, moreover, the only *in vitro* system for probiotic screening with documented clinical predictive validity. Foligné et al. (2007) demonstrated that PBMC-derived IL-10/IL-12 ratios predicted *in vivo* anti-inflammatory capacity in a murine TNBS-colitis model and that differences between blood donors did not prevent consistent strain ranking. This prediction was validated clinically by O'Mahony et al. (2005), confirmed at the human mucosal transcriptome level (van Baarlen et al., 2011), and translated into clinical trial strain selection in the PandA allergy prevention trial (Niers et al., 2005; Niers et al., 2009). No equivalent predictive validation chain exists for any cell line model, organoid system, or organ-on-a-chip platform in the context of probiotic immunomodulation (Papadimitriou et al., 2015; Klaenhammer et al., 2012).

The biological discriminatory power of the PBMC model across probiotic strains has been empirically quantified. Van Hemert et al. (2010) tested 42 *L. plantarum* strains with PBMCs from 3 donors and found IL-10 induction varied over a 14-fold range and IL-12 over a 16-fold range. The companion dendritic cell study by Meijerink et al. (2010) found even more dramatic variation: IL-10 ranged 39-fold and IL-12 ranged 600-fold across strains. Crucially, this study applied Random Forest gene-trait matching to identify six candidate genetic loci underlying the observed immunological differences. Notably, including the LamBDCA quorum sensing system and the plantaricin biosynthesis pathway, with gene deletion mutants confirming predictions, establishing the specific precedent for machine learning analysis of cytokine-induction data from probiotic-immune cell co-cultures that the present study extends to a multi-strain, multi-concentration, multi-preparation-type framework.

The substantial inter-donor variability in cytokine production capacity is genetically determined: Li et al. (2016) profiled PBMCs from 197 individuals and identified six genome-wide significant cytokine quantitative trait loci (QTLs), finding that cytokine production followed non-Gaussian or bimodal distributions with significantly increased inter-individual variability upon stimulation. Despite this variability, Foligné et al. (2007) and van Hemert et al. (2010) both demonstrated that relative strain rankings remain consistent across donors despite absolute value differences, validating the use of fold-change normalization and multiple donors as mitigation strategies. An important caveat noted by Kleiveland (2015) is that PBMCs "have a different composition, phenotype and activation status than cells found in intestinal tissue," lacking the epithelial barrier context and microbiome interactions present *in vivo*; the Foligné correlation, moreover, was established specifically for Gram-positive bacteria and should not be extrapolated to Gram-negative species. The PBMC model should therefore be understood as a validated comparative screening and ranking tool requiring complementary *in vivo* validation for translational conclusions.

Within the PBMC model specifically, dose-response data for probiotic immunomodulation remain strikingly scarce. Only three to four studies have attempted systematic multi-concentration characterization. Evrard et al. (2011) tested MOI 0.01–100 on dendritic cells and found strong dose-dependent DC maturation and cytokine induction with ~1,700 modulated genes at MOI 100 versus weak responses at MOI 0.01. Ghadimi et al. (2008) identified a dosage optimum of 10 bacteria per PBMC identical across all LAB tested, with bacterial genomic DNA contributing ~50% of effects dose-dependently. Hua et al. (2010) found that cytokine production decreased at 10^8^ CFU/mL compared to 10^6^–10^7^ CFU/mL, providing early evidence for non-linear, inverted-U dose-response kinetics consistent with the hormesis concept. None of these studies applied the scale of the present work (10 strains × 2 preparation types × 3 doses × 17 cytokines), used preparation-type-neutral flow cytometric input standardization, or applied machine learning analysis to the resulting dataset. The machine learning gap in probiotic immunological research is equally pronounced. Standard ML algorithms are routinely applied to microbiome data for disease classification — Li P et al. (2025) benchmarked nine algorithms across 83 cohorts and 20 diseases, and Wang and Liu (2020) compared classifiers across 29 benchmark microbiome datasets. ML has been applied to cytokine profiles for clinical disease stratification: Hong et al. (2022) demonstrated XGBoost classification of COVID-19 severity using IL-6 and IL-10 profiles with high discriminatory performance, establishing the precedent for ML on cytokine data in immunological contexts. Genomics-based probiotic classification has been developed by Sun et al. (2022), Orkkatteri Krishnan et al. (2025), and Shufang et al. (2024), achieving >95% accuracy for probiotic/non-probiotic binary classification from whole-genome features. Unsupervised ML has been applied to probiotic PBMC cytokine data by Coelho and Mossotto et al. (2020), who applied hierarchical clustering to cytokine profiles from PBMC stimulation assays targeting NOD2/TLR pathways, generating three distinct patient clusters — but their goal was patient stratification, not probiotic strain functional annotation. Wu et al. (2024) used unsupervised ML to differentiate 12 *Bifidobacterium* strains on an intestine-on-a-chip platform using organ-on-a-chip readouts rather than cytokine profiling. Charizani et al. (2024) described the first supervised ML model to predict *in vivo* probiotic potential from *in vitro* phenotypic features — but using cell surface properties (autoaggregation, hydrophobicity) in an aquatic/snail organism model, not cytokine profiles from human immune cells. Building on the ML-on-cytokine precedent established by Meijerink et al. (2010) in a single-species context, no published study has applied machine learning to classify probiotic strains by their immunomodulatory cytokine-induction fingerprint across a multi-strain, multi-dose, multi-preparation-type design in a primary human immune cell model.

The methodological weaknesses pervading the probiotic clinical trial literature compound all of the above gaps. Bafeta et al. (2018) found that among 384 probiotic RCTs, 28% reported no harms data, 80% did not report serious adverse events per group, and only 2% adequately reported all key safety components. McFarland et al. (2023) identified critical quality gaps across 206 probiotic systematic reviews, including inconsistent strain reporting and failure to account for dose differences. Suez et al. (2019) highlighted that strain-level evidence is often inappropriately extrapolated, safety is under-evaluated, and personalized immune responses are routinely ignored. Szajewska et al. (2025) concluded that no data causally link probiotic clinical benefits to microbiota protection or recovery. Against this backdrop, the present study was designed to provide the first systematic multi-strain experimental characterization of dose-response relationships in probiotic immunology, using primary human PBMC from a single healthy male Caucasian donor — a deliberate constraint accepted in order to resolve strain-level differences within one controlled experiment, at the explicit cost of population-level generalizability, and the reason this study is presented as a proof of concept — flow cytometric input standardization (AFU/TFU per ISO 19344:2015) applicable to both preparation types, 17-plex cytokine profiling, and machine learning-based functional strain classification. Ten Gram-positive probiotic strains were tested in both viable (AFU) and heat-treated (TFU) forms across three dose levels (10^5^, 10^6^, 10^7^ cells/well), with three technical replicates per condition. Primary aims: (i) to determine whether systematic dose-response relationships exist across the tested concentration range, characterizing their shape, magnitude, and strain-specificity; (ii) to establish, using concentration-matched AFU/TFU comparison, the dose at which heat-treated preparations achieve functional equivalence to viable preparations, operationalized as the Functional Equivalence Dose (FED^70^); and (iii) to determine whether machine learning can classify probiotic strains into functionally meaningful immunological phenotype groups based on their 17-plex cytokine-induction fingerprint.

**Section S2. Data Quality and Distribution Characteristics**

The final dataset comprised 192 observations across 17 cytokines (90 viable conditions + 90 heat-treated conditions + 12 negative controls). All 10 probiotic strains were represented equally (18 samples each), with balanced distribution across the three bacterial concentrations (60 samples per concentration level). The balanced factorial structure ensured equivalent statistical power for all comparisons, with each cell of the design containing exactly three technical replicates.

Substantial out-of-range values were observed for several cytokines, reflecting the wide dynamic range of immunological responses inherent to the experimental design. MIP-1β exhibited the highest proportion of values exceeding the upper quantification limit (33.9%), indicative of potent chemokine induction particularly by viable bacteria. IL-7 showed the highest proportion below detection (27.8%), consistent with constitutively low expression in this experimental system. Raw cytokine concentrations spanned several orders of magnitude and exhibited pronounced right-skewness characteristic of immunological data. Log^10^-transformation substantially improved distributional properties, with 16 of 17 cytokines showing increased Shapiro-Wilk p-values post-transformation. Coefficients of variation computed across the 60 experimental conditions (25.8% for MCP-1 to 588.0% for IL-1β) — a measure of condition-to-condition spread, not of assay precision or technical-replicate reproducibility reflected not merely technical noise but the substantial biological variability introduced by the 60-condition factorial design spanning two viability states and two orders of magnitude in bacterial concentration.

The overall pattern of immunomodulatory activity, visualised as mean log^2^ fold-change relative to the unstimulated control (Figure 2), revealed heterogeneity operating at three levels simultaneously: response magnitude varies markedly across strains; the concentration-output relationship differs systematically between strains and between viable and heat-treated forms; and the cytokine-level composition of the immunological response shifts as a function of both dose and preparation type. Three analytes — IL-8, MIP-1β, and TNF-α — reached or exceeded the ULOQ of the BioPlex assay in a subset of conditions and are fully annotated in Supplementary Figure 1. To assess the impact of imputed values on multivariate analyses, key results — including PCA component loadings, cluster assignments, and ML feature importance rankings — were verified to be qualitatively stable when MIP-1β and IL-7 were excluded from the analysis matrix. The principal conclusions reported below are therefore not dependent on the imputed values for these two analytes.

**Section S3. Dose-Response Profiles of Viable Preparations**

Six of the ten viable preparations exhibited dose-dependent cytokine increases consistent across multiple analytes, establishing dose-response as a generalizable principle of probiotic-PBMC interaction (Figure 6). These data provide, at the 10-strain panel scale, the systematic multi-concentration evidence base whose absence was documented by Ouwehand (2017), Shane et al. (2010), and EFSA (2012). The strongest relationship was observed for Strain B: IL-6 increased from 4,892-fold (±SD) at 10^5^ AFU/well to 17,455-fold at 10^6^ and 18,081-fold at 10^7:^ IL-1β increased from 1,806-fold at 10⁵ to 8,829-fold at 10⁶ and 58,409-fold at 10⁷ — a 32-fold amplification across the full two-log concentration range. Strain A displayed concordant monotonic escalation: IL-6 from 1,410-fold at 10⁵ to 3,614-fold at 10⁶ and 13,548-fold at 10⁷; G-CSF from 33-fold to 217-fold to 7,954-fold; IL-10 from 103-fold to 549-fold to 7,028-fold. Strains F, H, I, and L similarly exhibited monotonic profiles (≥85% of cytokines showing V5 ≤ V6 ≤ V7) with varying magnitudes. Strain G showed a predominantly monotonic pattern (71% of cytokines) with minor deviations in IL-6 and IL-10 at the highest concentration. The existence of a minimum effective concentration and an optimal dose range where response scaling is steepest are directly apparent, providing the pre-clinical dose-characterization evidence that regulatory bodies require and that the field has been unable to supply.

**Section S4. Non-Monotonic Dose-Response Profiles: Bell-Shaped Kinetics**

Two strains — D and E — exhibited non-monotonic, bell-shaped kinetics with peak activity at 10^6^ AFU/well and measurable, multi-cytokine attenuation at 10^7^, consistent across technical replicates and consistent with the inverted-U dose kinetics reported by Hua et al. (2010) and the dosage optimum identified by Ghadimi et al. (2008). For Strain E: IL-6 increased from 1,379-fold at 10⁵ to a peak of 2,383-fold at 10⁶ before declining to 1,778-fold at 10⁷ (−25%); G-CSF showed the most pronounced pattern (1,055 → 1,480 → 246-fold, −83% from peak); IL-10 followed concordantly (308 → 377 → 95-fold). Strain D displayed a structurally parallel pattern across IL-6 (490 → 1,185 → 598-fold), G-CSF (61 → 411 → 263-fold), IL-10 (35 → 72 → 49-fold), with the peak uniformly at 10^6^. IFN-γ showed an inverse pattern for both strains, declining monotonically (Strain E: 52 → 44 → 24-fold; Strain D: 26 → 17 → 6-fold), indicating cytokine-specific rather than uniform attenuation. A critical dissociation was observed: heat-treated preparations for both strains showed stable or continuing activity at 10^7^ TFU/well for several analytes, arguing against non-specific cytotoxicity and in favour of an active-cell-dependent mechanism. The mechanistic interpretation of this dissociation is discussed in Section 4.

**Section S5. Strain C: A Strongly Viability-Dependent Immunomodulatory Profile**

Strain C occupied a singular position in the dataset. The viable form induced modest responses with cytokine-specific dose patterns: IL-6 showed no directional trend (1,142-, 800-, 1,014-fold across three dose points), while IL-10 displayed a progressive increase (21-, 57-, 180-fold). Overall immunostimulatory activity was substantially lower than high-magnitude strains such as B and A. The heat-treated form showed minimal induction across the cytokine panel at all concentrations: IL-6 (0.7, 0.9, 2.7-fold), IL-10 (0.2, 0.1, 0.4-fold), IL-1β (0.3, 0.3, 0.6-fold), G-CSF (<0.1-fold). At 10⁷ TFU/well — where heat-treated Strains B, H, and E elicit thousands-fold inductions — Strain C heat-treated preparations remained near baseline for 12 of 14 quantifiable analytes. This establishes that the strong attenuation of activity following heat treatment is a strain-specific biological property, not a generic consequence of microbial material at a given concentration, directly demonstrating that postbiotic activity cannot be assumed without explicit functional characterization of the specific heat-treated preparation.

**Section S6. Unsupervised k-means partitioning of the full dataset**

*This analysis is descriptive and does not bear on the study's primary questions.*

Both the silhouette score and the Calinski-Harabasz index identified k = 2 as the optimal cluster number across k = 2 to 10, indicating that the dominant partition in the data corresponds to a binary variable, consistent with the viability-state factor. K-means clustering at k = 2 revealed a strong but imperfect association between cluster membership and bacterial viability status. The partition, computed on the 60 condition means, comprised a larger, lower-magnitude cluster (n = 45; 40.0% heat-treated) and a smaller cluster predominantly composed of heat-treated conditions (n = 15; 80% heat-treated). The imperfect alignment between clusters and viability states is informative: the viable samples assigned to the predominantly heat-treated cluster were drawn exclusively from the lowest concentration (10⁵ AFU/well), where response magnitude approaches that of heat-treated preparations, while heat-treated samples assigned to the predominantly viable cluster were concentrated at the higher doses. The partition therefore tracks response magnitude rather than viability state as such, which is consistent with the dominance of a single variance axis reported in Section 3.10 of the main text.

**Section S7. Comparative classifier performance for viability status**

*This section documents the basis for the AUC-ROC value cited in the main text.*

Four classification algorithms were evaluated for binary discrimination of viable from heat-treated responses on 16-cytokine profiles (IL-8 excluded), using the fixed hyperparameters declared a priori in the deposited configuration file. Classification was performed on condition means (n = 60) under leave-one-out cross-validation. Random Forest achieved AUC-ROC 0.702 (accuracy 63.3%), XGBoost 0.664 (65.0%), Logistic Regression 0.674 (61.7%) and the radial-basis-function Support Vector Machine 0.517 (50.0%), the last of these being indistinguishable from chance (Supplementary Figure 3). The tree-based ensemble returned the highest AUC-ROC, but the differences between classifiers are small: McNemar's exact test did not establish a significant difference between the Random Forest and either the Support Vector Machine (p = 0.12) or Logistic Regression (p = 1.00), the latter two differing by a single prediction out of 60. No claim of algorithmic superiority is made.

Feature importance rankings were broadly concordant between the tree-based and linear classifiers, with IL-6 the highest-ranked discriminator in each. The Support Vector Machine performed at chance and its ranking is therefore not treated as corroborating evidence. Because these performance estimates derive from a single donor and from condition means rather than independent biological replicates, they describe the separability of the two preparation types within this experiment and are not estimates of generalisation to new donors or runs.

**Section S8.** Bayesian and pathway-level description

The Bayesian framework adds probabilistic quantification to this viability comparison. While live conditions achieved P(immunostimulatory) ranging from 0.64 to 0.97, heat-treated conditions at 10⁵ TFU/well for Strains C and I yielded P values as low as 0.19, identifying conditions where immunostimulation is genuinely uncertain rather than merely reduced in magnitude. This probabilistic view complements the frequentist fold-change analysis and provides actionable dose thresholds. Pathway mapping further demonstrates that the viability effect is not pathway-selective: all twelve signaling cascades show significantly higher activation in live conditions, confirming a generalized amplification mechanism consistent with continuous MAMP release and active metabolite production by viable bacteria.

**Section S9.** Extended background: market context, the regulatory record and the enumeration crisis

This re-examination has gained formal recognition through the International Scientific Association for Probiotics and Prebiotics definition of postbiotics as “preparations of inanimate microorganisms and/or their components that confer a health benefit on the host” (Salminen et al., 2021), which explicitly requires deliberate inactivation, a defined source microorganism, and demonstrated health benefit. The concept that probiotic immunomodulatory activity can persist independently of viability has been reviewed extensively (Taverniti and Guglielmetti, 2011). The clinical evidence supporting this paradigm has grown substantially. Depommier et al. (2019) demonstrated in a randomized, double-blind, placebo-controlled trial that pasteurized *Akkermansia muciniphila* significantly improved insulin sensitivity, reduced insulinemia, and lowered total cholesterol, while the live form did not reach significance on primary endpoints. A systematic review of 40 randomized controlled trials encompassing 3,913 participants found that modified probiotics were not significantly different from living probiotics in 86% of preventive and 69% of treatment trials, with treated bacteria actually outperforming live preparations in 15% of treatment trials (Zorzela et al., 2017). The largest randomized controlled trial of a heat-treated bacterium for irritable bowel syndrome further reinforced this evidence: Andresen et al. (2020) demonstrated that heat-treated *Bifidobacterium bifidum* MIMBb75 achieved a 34% versus 19% placebo response rate in 443 patients. These findings raise a fundamental question: if heat-treated bacteria can match or exceed the efficacy of live preparations, then the requirement for viability—and the entire construct of “adequate amounts” based on CFU enumeration of viable cells—requires reconsideration.

The global probiotic market, valued at over 60 billion US dollars, rests on a scientific foundation that leading researchers increasingly regard as insufficient (Suez et al., 2019). Every probiotic product on the market declares a dose, yet none can provide rigorous evidence that the declared colony-forming unit count—whether one billion, ten billion, or two hundred billion—was selected based on biologically defined effective concentrations rather than manufacturing feasibility and competitive positioning. The International Scientific Association for Probiotics and Prebiotics defined probiotics as “live microorganisms that, when administered in adequate amounts, confer a health benefit on the host” (Hill et al., 2014), yet what constitutes “adequate amounts” remains remarkably poorly characterized at the strain level. The foundational FAO/WHO consensus encoded the phrase “adequate amounts” without any operational framework to define it (FAO/WHO, 2001), and the widespread assumption that a minimum of one billion colony-forming units (CFU) is required for efficacy has become an industry standard despite the absence of rigorous dose-ranging evidence for most strains and endpoints (Ouwehand, 2017). The regulatory consequences of this evidence vacuum are stark: the European Food Safety Authority has rejected every probiotic health claim submitted—over 100 applications—partly due to insufficient dose characterization (EFSA, 2012), the United States Food and Drug Administration considers probiotic disease claims as drug uses requiring full Investigational New Drug and New Drug Application pathways with no probiotic health claim approved to date, and the ICH E4 guideline mandates dose-response assessment as “an integral component of drug development” with a minimum of three dosage levels—a standard essentially absent in the probiotic field. Ouwehand (2017) reviewed seven meta-analyses spanning major probiotic endpoints and found no clear dose-response relationship for immune markers, bowel function, or general health outcomes. Only antibiotic-associated diarrhea showed a threshold effect near 1010 CFU per day. Even in vulnerable populations where dose optimization would carry the greatest clinical consequence, the evidence remains thin: Rath C et al. (2023) found limited dose-response evidence in preterm infants, with one randomized controlled trial showing no significant differences between high- and low-dose groups. The International Scientific Association for Probiotics and Prebiotics working group explicitly criticized the field, noting that “multiple doses are needed in order to elucidate the presence of dose-dependence” and that “single-dose studies are rarely acceptable” (Shane et al., 2010). The World Gastroenterology Organisation acknowledges that effective probiotic doses range from 108 CFU per day for *Bifidobacterium longum* 35624 in irritable bowel syndrome to 450 billion CFU thrice daily for VSL#3—spanning a greater than 1,000-fold range—yet almost no studies have characterized the dose-response curve shape (WGO, 2023). In the only published five-dose-level human immune study, Christensen et al. (2006) found no significant immune changes at any dose level from 10⁸ to 10¹¹ CFU per day in 71 healthy adults, illustrating both the scarcity of dose-ranging studies and the challenge of translating in vitro findings to clinical immunological endpoints. Despite these admonitions, the vast majority of published probiotic studies employ a single dose without pharmacological justification, a practice that would be unacceptable in pharmaceutical development where ICH E4 guidelines mandate dose-response assessment with at least three dosage levels plus placebo. Health Canada takes a more progressive strain-specific approach but still requires evidence to support the daily dose for each strain—a requirement that presupposes the existence of dose-response data that, for immunological endpoints, the field has largely failed to generate. A second foundational assumption—that colonization of the gut is required for efficacy—has been systematically challenged. Zmora et al. (2018) used invasive endoscopic sampling to demonstrate that mucosal probiotic colonization is highly person-, region-, and strain-specific, and that stool analysis does not reflect mucosal colonization status. The companion study by Suez et al. (2018) showed that probiotic colonization after antibiotics actually delayed indigenous microbiome reconstitution versus spontaneous recovery or autologous fecal microbiota transplantation. Kristensen et al. (2016) found no evidence that probiotics alter fecal microbiota composition in healthy adults across seven randomized controlled trials, suggesting that effects derive from transient metabolic activity and immune modulation rather than colonization. Suez et al. (2019) further highlighted that strain-level evidence is often inappropriately extrapolated across species, safety is under-evaluated, and personalized immune responses are routinely ignored. If colonization is neither reliable nor necessary, then the viability requirement embedded in the canonical definition itself demands re-examination.

This reconsideration is further complicated by the quantification crisis pervading probiotic research. Colony-forming unit plate counting, the industry standard, systematically underestimates viable cell populations by missing viable-but-non-culturable cells (Davis, 2014). A landmark interlaboratory study across 244 laboratories demonstrated that optical density at 600 nm calibrated against CFU produced values more than an order of magnitude different from flow cytometry ground truth, with the same optical density reading yielding CFU estimates spanning a 3.3-fold range between instruments (Beal et al., 2020). Flow cytometry enumeration has been shown to detect approximately 70% more viable cells than plate counting for the same product (Visciglia et al., 2022), with precision nearly twice that of conventional methods: relative standard deviation of 6.70% versus 12.52% for plate counting (Tracey et al., 2023). Reproducibility between standardized plate count methods ranges from 0.5 to 1 log, meaning results can differ up to 10-fold between laboratories (Foglia et al., 2020). The discrepancy widens with storage time as cells transition to the viable-but-non-culturable state: Gorsuch et al. (2019) showed that flow cytometric and quantitative polymerase chain reaction counts were similar and substantially higher than plate counts at later storage times, rendering CFU-based dosing increasingly unreliable for aged preparations. Critically, CFU methodology is entirely inapplicable to heat-treated preparations by definition. A 2024 Frontiers in Microbiology study (Bolzon et al., 2024) developed the first optimized flow cytometric method for tyndallized bacterial cells using TO/PI double staining. The regulatory adoption of flow cytometric total fluorescent unit enumeration for pasteurized *Akkermansia muciniphila*—the first next-generation bacterium to receive EFSA novel food approval (EFSA, 2021)—established the regulatory precedent for culture-independent quantification. Flow cytometry enumeration, standardized through ISO 19344 and validated across 15 laboratories in 5 countries, provides the methodological foundation for quantifying both viable and heat-treated preparations in equivalent units, which is what enabled the concentration-matched comparison made here of live and heat-treated bacteria at matched concentrations.

**Section S10.** Composition of the inter-cytokine correlation clusters

The correlation matrix resolved two major clusters. The first comprised TNF-α, IL-1β, IL-6, IFN-γ, IL-2, and IL-12p70, representing coordinated pro-inflammatory and Th1-type responses driven by monocyte/macrophage and T cell activation. The second cluster included IL-4, IL-5, IL-10, and IL-13, reflecting Th2-type adaptive immune responses. G-CSF and GM-CSF formed a partially independent sub-cluster associated with myeloid cell proliferation and differentiation. IL-8 and MIP-1β correlated moderately with each other (ρ = 0.53) and with the pro-inflammatory cluster. MCP-1 showed the most independent expression pattern, with substantially lower correlations to both major clusters (mean ρ with pro-inflammatory cluster = 0.27; mean ρ with Th2 cluster = 0.16), consistent with its unique viability-independence demonstrated in Section 3.2 and its operation through distinct regulatory pathways responding to bacterial structural presence rather than metabolic activity. Analysis of cytokine co-regulation profiles across doses revealed that increasing concentration alters the qualitative composition of the immunological signature in a strain-specific manner (Figure 8). For Strain B, non-isometric expansion was observed: IL-6, IL-1β, IL-10, and G-CSF scaled dramatically while IFN-γ, IL-7, and IL-17 showed modest dependence. Strain E showed qualitative remodelling at 107 AFU/well: G-CSF collapsed in the viable form (1,480 → 246-fold) while the heat-treated form produced 2,059-fold — an analyte dominance inversion visible only through multiplex profiling across both forms and the full concentration range. It is noted that the correlation matrix reflects the full dataset spanning all viability states and dose levels, and therefore captures global response amplitude as a major co-variation source. Cytokine pairs that appear strongly correlated at the dataset level may not be co-regulated within individual conditions — a distinction relevant for mechanistic interpretation.

**Section S11. Per-strain composition of the functional groups**

The fourth pillar of this study addresses a methodological opportunity rather than a limitation: the use of machine learning to extract information from well-structured experimental data that goes beyond the original experimental hypothesis. The ML analysis reported here is, to our knowledge, the first application of supervised and unsupervised classification to probiotic strains based on their immunomodulatory cytokine-induction phenotype. The classification accuracies reported reflect descriptive pattern recognition within a single-donor, technical-replicate dataset and should not be interpreted as inferential estimates of generalization to novel donors or independent experimental runs. Charizani et al. (2024) described the first supervised ML model for probiotic potential prediction from in vitro phenotypic features, but used cell surface properties in an aquatic model. Coelho and Mossotto et al. (2020) applied hierarchical clustering to PBMC NOD2/TLR stimulation cytokine data for patient stratification — the closest methodological precedent in the immunological context, applied to a different classification goal. Wu et al. (2024) differentiated 12 *Bifidobacterium* strains by ML on intestine-on-a-chip readouts rather than cytokine profiles. The present study bridges these parallel trajectories: genomic ML (Orkkatteri Krishnan et al., 2025; Sun et al., 2022; Shufang et al., 2024), clinical cytokine ML (Hong et al., 2022; Li P et al., 2025), and functional probiotic phenotyping (van Hemert et al., 2010; Meijerink et al., 2010) — extending the Random Forest gene-trait matching of the latter into the immunological phenotype space itself.

More provocatively, the machine learning approach revealed functional phenotypes that were not part of the original experimental question. Three distinct immunomodulatory groupings were obtained by unsupervised clustering, without prior functional annotation (the Th1 and Th2 cytokine sets used to compute the polarisation scores were, by contrast, defined a priori): high-magnitude/Th1-biased (Strains B, A), moderate with variable polarization (Strains E, F, G, H, I, L), and low-magnitude (Strains C, D). The high cluster classification accuracy (83.3% LOO-CV) reflects the internal separability of these groups within this dataset; because the clusters were defined from the same data on which the classifier was trained, this metric should not be interpreted as the expected accuracy for classifying novel strains into these phenotype groups. The identification of these phenotypes among just 10 strains carries a sobering implication for the field. McFarland et al. (2023) documented that probiotic systematic reviews and meta-analyses continue to group strains of the same species as interchangeable. Our data demonstrates that immunomodulatory phenotypes within a small collection range from strongly pro-inflammatory to anti-inflammatory. Pooling such strains in a meta-analysis is pharmacologically equivalent to combining clinical trials of agonists and antagonists acting on the same target and concluding that the drug class has “no effect.”

Z-score heatmap and functional radar plot analysis revealed distinct immunomodulatory profiles. The strain groupings were obtained by unsupervised clustering without prior functional annotation; the Th1-associated and Th2-associated cytokine sets used to compute the polarisation scores were, by contrast, defined a priori (Section 2.7.2). In this single-donor model, Strain B exhibited the most potent immunostimulatory profile: TNF-α z-score = +0.51, IL-1β = +1.88, IL-6 = +1.90, IFN-γ = +1.68, with the strongest Th1 bias among high-magnitude strains. Strain A showed a similar but less pronounced pro-inflammatory profile (TNF-α z = +0.51, IFN-γ = +1.12, IL-6 z = +1.62).

Strains E, F, G, H, I, and L clustered together with moderate pro-inflammatory responses and diverse Th1/Th2 polarization, with Strains G, H, and I showing Th2-biased profiles and Strains E and F showing near-balanced activation. Strains C and D exhibited low-magnitude profiles; Strain D showed a balanced Th1/Th2 profile while Strain C showed a markedly viability-dependent profile, with substantially attenuated immunomodulatory activity in the heat-treated form, consistent with its unique thermolabile phenotype. The absolute z-score values reported reflect this donor's immune background and are expected to vary in absolute magnitude across donors, though relative rankings are anticipated to be preserved.

The inter-strain variation for individual cytokines was substantial: IL-12p70 varied more than 40-fold between the highest-producing strain (B) and the lowest (D) at 10⁷ AFU/well (viable preparations), consistent with the 16-fold variation reported by van Hemert et al. (2010) for *L. plantarum* strains. Functional cluster classification (3-class, chance 33.3%) achieved 83.3% LOO partition-recovery consistency — 2.50× above chance. Because the three clusters were defined by unsupervised hierarchical clustering of the same cytokine data used to train and evaluate this classifier, this metric reflects internal consistency only and should not be interpreted as expected accuracy for classifying novel strains into these phenotype groups. Binary preparation-type classification (viable vs heat-treated, chance 50%) achieved 63.3% LOO accuracy.

**Section S12. Replicate-level variance decomposition**

*The k-means partition is reported once, in Section S6.*

To characterize the global variance structure of the complete dataset including technical replicates, PCA was performed on all 180 treatment observations — complementing the condition-mean PCA reported in Section 3.9 which addressed strain-level fingerprint organization. Principal component analysis on the full dataset (180 treatment observations × 17 analytes — IL-8 is retained here, unlike in the supervised analyses, because the unsupervised decomposition is descriptive and no class label is being predicted; RobustScaler-standardized log-transformed data) revealed a highly concentrated variance structure. The first principal component explained 74.7% of total variance, implying that a single dominant biological process — overall immune activation magnitude — drives the majority of observed variation. PC1 was loaded by multiple pro-inflammatory and adaptive cytokines, with IL-5, IL-2, IL-4, IL-17A and IL-1β among the strongest contributors (each ≈ 0.29–0.30), with MCP-1 dominating the second component (loading ≈ 0.79), reflecting the coordinated nature of the cytokine response rather than dominance of a single analyte. PC2 (9.4% variance) was dominated by MCP-1 (loading = 0.774), representing the chemokine axis orthogonal to overall immune activation captured by PC1. PC3 (5.3% variance) captured additional myeloid activation variance associated with GM-CSF and G-CSF. Cumulatively, three components explained 89.5% and five components explained 92.7% of total variance, demonstrating that the 17-dimensional cytokine space can be effectively represented by a low-dimensional manifold. Score plots coloured by viability status demonstrated clear separation between viable and heat-treated samples along the PC1 axis: viable bacteria samples occupied the positive PC1 space (higher immune activation), while heat-treated samples were displaced toward the negative end. Within each viability group, concentration-dependent gradients were visible, with 107 AFU/well conditions positioned at higher PC1 values than 105 conditions. This visual separation is described quantitatively by the classifier comparison reported (Section 3.12).

**Section S13. Dose-response evidence for the structural-signal interpretation**

This interpretation is directly supported by our dose-response data. Heat-treated preparations, whose nominal concentration is not increased by replication during the experiment, demonstrated significantly stronger Spearman dose-response correlations (mean within-strain ρ up to 1.00, attained by IL-4; 0.97 for MIP-1β and G-CSF) than viable preparations for 13 of 17 cytokines — despite inducing lower absolute responses. This is consistent with — though not proven by — the hypothesis that the input variable is more accurately controlled for heat-treated than for viable preparations. The weaker dose-response correlation in viable conditions is most parsimoniously explained, we argue, as a potential artefact of uncontrolled bacterial proliferation compressing the effective dose range, rather than evidence of a fundamentally different dose-response biology. Alternative explanations — including greater intrinsic biological variability in the live-bacteria response — cannot be excluded without endpoint enumeration data. This finding has a direct implication for the interpretation of all published probiotic PBMC co-culture studies that compare viable bacteria across dose levels without endpoint enumeration: their dose-response data are potentially confounded by unquantified within-experiment concentration drift.

The practical implication is methodological and productive: heat-treated preparations provide a more experimentally tractable model for dose-response characterization because their concentration is not subject to bacterial replication during the experiment, although lysis, aggregation and progressive structural degradation of heat-treated cells were not measured and cannot be excluded. Salminen et al. (2021)’s postbiotic definition thus gains an unexpected dimension: postbiotics may offer a more tractable experimental system for controlled pharmacological investigation of dose-response, because the input concentration is not altered by replication during co-culture. This finding aligns with and extends the clinical postbiotic literature — the landmark Depommier et al. (2019) trial showed pasteurized *Akkermansia muciniphila* outperforming its live counterpart on primary metabolic endpoints, Zorzela et al. (2017) found equivalence in 86% of preventive trials, and Andresen et al. (2020) demonstrated clinical efficacy of heat-treated *B. bifidum* MIMBb75 in irritable bowel syndrome — by adding a mechanistic layer: the apparent immunological superiority of live preparations may be partly illusory, reflecting experimental artefacts from bacterial proliferation rather than genuine pharmacological differences.

**Section S14. Low-concentration response, regulatory reading and strain-specific dose sensitivity**

The detection of immunologically meaningful responses even at the lowest concentration (105 AFU/well) indicates that in vitro immunological activity was not confined to the higher concentrations tested; no inference about oral dose is drawn. This is distinct from the widespread assumption that a minimum of approximately 106 bacteria per milliliter is required for immune effects. While responses at 105 AFU/well were quantitatively lower than at higher concentrations, they were consistently above negative control levels for most cytokines in both viability states. This observation may have practical significance for the postbiotic field, where delivered doses may be lower than those achievable with viable preparations due to formulation constraints.

From a regulatory perspective, these data demonstrate that the in vitro PBMC co-culture system, when combined with appropriate bacterial enumeration and statistical analysis, can generate the type of dose-response evidence that regulatory bodies require but the field has largely failed to produce. The EFSA requirement that clinical evidence must be conducted “at the same dosage” as the marketed product, and the ICH E4 mandate for at least three dosage levels in dose-finding studies, illustrate standards that the probiotic literature has rarely approached. The concentration-matched AFU/TFU comparison enabled by flow cytometric quantification further allows the determination of a Functional Equivalence Dose — the concentration at which heat-treated preparations achieve a defined percentage of the viable preparation’s immunological output. This FED concept, adapted from the bioequivalence acceptance framework (EMEA, 2010), offers a candidate strain- and endpoint-specific screening metric which, if validated across donors, could contribute to postbiotic dose justification.

The strain-specific dose-response heatmaps reveal an additional layer of complexity not captured by aggregate analysis. Strains A and B maintain robust dose-dependency in both viability states, while Strain E shows near-absent dose-response in live conditions, with the majority of cytokines showing non-monotonic or flat patterns (ρ = 0 or ρ = −0.5)— suggesting — if the proliferation hypothesis holds — that dose compression may be strain-dependent, potentially reflecting differences in growth kinetics under co-culture conditions. For heat-treated preparations, the generally stronger and more monotonic dose-response, which varied in magnitude between strains suggests, subject to multi-donor confirmation, that dose-optimization studies may benefit from using heat-treated preparations to obtain interpretable pharmacological data unconfounded by inter-strain variation in growth rate.

**Section S15. Machine-learning reading of MCP-1 viability-independence**

From an ML perspective, the viability-independence of MCP-1 is equally significant: despite showing no differential expression between viable and heat-treated conditions globally, MCP-1 ranked as the third most important feature for strain classification (importance = 0.090). This dissociation — between MCP-1’s failure to discriminate viability states and its contribution to strain identity discrimination — illustrates a core principle of ML-informed multiplex analysis: the informativeness of a cytokine depends entirely on the classification question. An analyte uniformly insensitive to one variable may be highly informative for another. This finding could not have been identified through hypothesis-driven, single-analyte screening and demonstrates the value of unbiased multiplex ML feature selection.

**Section S16. Bacterial enumeration: flow cytometry versus colony counting, and the regulatory precedent**

The choice of bacterial enumeration method is not a technical footnote — it is a determinant of whether dose-response data are interpretable. This study employed flow cytometry enumeration according to ISO 19344, with results expressed in active fluorescent units for viable preparations and total fluorescent units for heat-treated preparations. Three dimensions of this choice merit emphasis. First, flow cytometry provides substantially greater precision than plate counting: Tracey et al. (2023) quantified the relative standard deviation of flow cytometry at 6.70% versus 12.52% for conventional plate counting (P = 0.0002), with viable cell recovery of 101.8 ± 6.95% by flow versus 81.37 ± 16.03% by plate count. In practical terms, the three concentration levels in our study are known with approximately 7% coefficient of variation; with plate counting, the same **nominal concentrations could vary by 15–**30%, substantially reducing the statistical power to detect concentration-dependent effects. The Christensen et al. (2006) null result in a five-dose human study may thus reflect dose uncertainty as much as genuine dose-independence.

Second, and critically for the live versus heat-treated comparison at the heart of this study, flow cytometry is the only enumeration method capable of verifying dosing equivalence between the two viability states. Colony-forming unit plate counting yields zero for heat-treated preparations by definition; optical density measurements cannot distinguish intact from lysed cells. Flow cytometry, by detecting individual intact fluorescently labelled cells regardless of metabolic activity, provides direct verification that equivalent numbers of intact bacterial cells are present in both preparations — a standard that CFU-based approaches fundamentally cannot meet for heat-treated material.

Third, the regulatory significance of this methodological choice is growing. The European Food Safety Authority approved pasteurized *A. muciniphila* as a novel food in 2021 based on total fluorescent unit enumeration by flow cytometry rather than colony-forming unit counting (EFSA, 2021), establishing a precedent that may reshape how probiotic and postbiotic doses are reported. We note with concern that the vast majority of published probiotic immunomodulation studies continue to rely on colony-forming unit plate counting or optical density-based estimates. When the input variable of a dose-response experiment is known only within an order of magnitude, the experiment cannot distinguish dose-dependent from dose-independent effects — regardless of statistical sophistication applied to the output.

The practical framework that emerges is straightforward: flow cytometry-enumerated bacteria at defined concentrations, 24-hour PBMC co-culture, multiplex cytokine quantification, and machine learning classification constitute a comprehensive immunomodulatory characterization pipeline completable within approximately 48 hours from bacterial preparation to functional annotation — the co-culture and readout steps require approximately 48 hours once bacterial cultures are available.

While viable conditions achieved P(immunostimulatory) between 0.64 and 0.97, heat-treated conditions at 10⁵ TFU/well for Strains C and I yielded values as low as 0.19, identifying conditions in which immunostimulation is genuinely uncertain rather than merely reduced in magnitude. This probabilistic view complements the fold-change analysis and provides a descriptive summary of response breadth across concentrations; as noted in Section 2.7.3 of the main text, the binomial model assumes independence between analytes, which the observed inter-cytokine correlations do not satisfy, so the posteriors are not an independent confirmation of the frequentist result. Pathway mapping shows higher aggregate activity in viable conditions for all 12 pathway categories. This is a visual aggregation rather than a pathway-level statistical test, and is consistent with, but does not demonstrate, generalised amplification.

**Supplementary Figure 1.** Complete 17-analyte cytokine dataset. All 17 BioPlex analytes including IL-8, MIP-1β, and TNF-α. Black cells with white text ('>UL'): values exceeding the upper limit of quantification (assay upper limits of quantification, expressed as sample concentrations after correction for the applied dilution factor: IL-8 1.97 x 10^4 pg/mL; MIP-1β 2.68 x 10^4 pg/mL; TNF-α 6.24 x 10^3 pg/mL), present in 11, 18, and 14 of 60 conditions respectively. Row organisation as Figure 2: strains A–L, each with viable (V5, V6, V7) followed by heat-treated (HT5, HT6, HT7) preparations. Colour scale: diverging RdYlBu, capped at log₂ = −5 (lower) and +12 (upper). (ULOQ values expressed as absolute concentrations in pg/mL as reported by Bio-Plex Manager software; fold-change values derived from these imputed concentrations are correspondingly extreme and are excluded from primary heatmap analyses)

**Supplementary Figure 2.** Distribution of log₂ fold-change (versus unstimulated control) for viable (AFU) versus heat-treated (TFU) preparations across 30 strain×dose conditions per group, shown per cytokine. Split violins show the full distribution; box = IQR (Q1–Q3), horizontal line = median, points = individual conditions.

**Supplementary Figure 3.** Viability-classification performance across four machine-learning algorithms (leave-one-out cross-validation AUC-ROC). Random Forest achieved the highest performance (AUC 0.702); SVM-RBF performed near chance (0.517). Dashed line: chance level (0.5).

**Supplementary Figure 4.** Complete per-strain dose-response profiles, Strains A–G. Fold-change (relative to unstimulated control) for all 17 analytes at 10⁵, 10⁶ and 10⁷ units per well, shown separately for viable (AFU, blue) and heat-treated (TFU, orange) preparations. This panel and Supplementary Figure 5 together carry the full ten-strain set summarised in main-text Figure 6, which shows representative strains only.

**Supplementary Figure 5.** Complete per-strain dose-response profiles, Strains H–D. Plotted as Supplementary Figure 4; together the two panels cover all ten strains.

| Strain Code | Strain Designation | Deposit Number | Strain Species |
| --- | --- | --- | --- |
| A | BS10 | DSM 35213 | *Bifidobacterium animalis subsp. lactis* |
| B | LP14 | DSM 33401 | *Lactiplantibacillus plantarum* |
| C | BR06 | LMG P-34167 | *Bifidobacterium breve* |
| D | BB10 | DSM 33678 | *Bifidobacterium bifidum* |
| E | LR04 | DSM 16605 | *Lacticaseibacillus rhamnosus* |
| F | LF26 | DSM 33402 | *Limosilactobacillus fermentum* |
| G | LKF03 | CCOS 2122 | *Lentilactobacillus kefiri* |
| H | LJO02 | DSM 33828 | *Lactobacillus johnsonii* |
| I | LA12 | LMG P-34340 | *Lactobacillus acidophilus* |
| L | LRE11 | DSM 33827 | *Limosilactobacillus reuteri* |

**Supplementary Table 1.** Correspondence between the blinded strain codes (A–L) used throughout the manuscript and the probiotic strain designations.

**Supplementary References**

Abraham, R. T., and Weiss, A. (2004). Jurkat T cells and development of the T-cell receptor signalling paradigm. *Nat. Rev. Immunol.* 4, 301–308. doi: 10.1038/nri1330

Andresen, V., Gschossmann, J., and Layer, P. (2020). Heat-inactivated *Bifidobacterium bifidum* MIMBb75 (SYN-HI-001) in the treatment of irritable bowel syndrome. *Lancet Gastroenterol. Hepatol.* 5, 658–666. doi: 10.1016/S2468-1253(20)30056-X

Bafeta, A., Koh, M., Riveros, C., and Ravaud, P. (2018). Harms reporting in randomized controlled trials of interventions aimed at modifying microbiota. *Ann. Intern. Med.* 169, 240–247. doi: 10.7326/M18-0343

Bar-Ephraim, Y. E., Kretzschmar, K., and Clevers, H. (2020). Organoids in immunological research. *Nat. Rev. Immunol.* 20, 279–293. doi: 10.1038/s41577-019-0248-y

Beal, J., Farny, N. G., Haddock-Angelli, T., Selvarajah, V., Baldwin, G. S., Buckley-Taylor, R., et al. (2020). Robust estimation of bacterial cell count from optical density. *Commun. Biol.* 3:512. doi: 10.1038/s42003-020-01127-5

Bolzon, V., Bulfoni, M., Pesando, M., Nencioni, A., and Nencioni, E. (2024). A streamlined workflow for a fast and cost-effective count of tyndallized probiotics using flow cytometry. *Front. Microbiol.* 15:1389069. doi: 10.3389/fmicb.2024.1389069

Bosshart, H., and Heinzelmann, M. (2016). THP-1 cells as a model for human monocytes. *Ann. Transl. Med.* 4:438. doi: 10.21037/atm.2016.08.53

Charizani, E., Dushku, E., Kyritsi, M., Metallinou, E. T., Karathodorou, A., Amanetidou, E., et al. (2024). Predicting the immunomodulatory activity of probiotic lactic acid bacteria using supervised machine learning in a *Cornu aspersum* snail model. *Fish Shellfish Immunol.* 152:109788. doi: 10.1016/j.fsi.2024.109788

Christensen, H. R., Larsen, C. N., Kaestel, P., Rosholm, L. B., Sternberg, C., Michaelsen, K. F., et al. (2006). Immunomodulating potential of supplementation with probiotics: a dose-response study in healthy young adults. *FEMS Immunol. Med. Microbiol.* 47, 380–390. doi: 10.1111/j.1574-695X.2006.00109.x

Clevers, H. (2016). Modeling development and disease with organoids. *Cell* 165, 1586–1597. doi: 10.1016/j.cell.2016.05.082

Coelho, T., and Mossotto, E. (2020). Immunological profiling of paediatric inflammatory bowel disease using unsupervised machine learning. *J. Pediatr. Gastroenterol. Nutr.* 70, 833–840. doi: 10.1097/MPG.0000000000002719

Davis, C. (2014). Enumeration of probiotic strains: review of culture-dependent and alternative techniques to quantify viable bacteria. *J. Microbiol. Methods* 103, 9–17. doi: 10.1016/j.mimet.2014.04.012

de Jager, W., te Velthuis, H., Prakken, B. J., Kuis, W., and Rijkers, G. T. (2003). Simultaneous detection of 15 human cytokines in a single sample of stimulated peripheral blood mononuclear cells. *Clin. Diagn. Lab. Immunol.* 10, 133–139. doi: 10.1128/cdli.10.1.133-139.2003

Depommier, C., Everard, A., Druart, C., Plovier, H., Van Hul, M., Vieira-Silva, S., et al. (2019). Supplementation with *Akkermansia muciniphila* in overweight and obese human volunteers. *Nat. Med.* 25, 1096–1103. doi: 10.1038/s41591-019-0495-2

Dijkstra, K. K., Cattaneo, C. M., Weeber, F., Chalabi, M., van de Haar, J., Fanchi, L. F., et al. (2018). Generation of tumor-reactive T cells by co-culture of peripheral blood lymphocytes and tumor organoids. *Cell* 174, 1586–1598. doi: 10.1016/j.cell.2018.07.009

Duffy, D., Rouilly, V., Libri, V., Hasan, M., Beitz, B., David, M., et al. (2014). Functional analysis via standardized whole-blood stimulation systems defines the boundaries of a healthy immune response to complex stimuli. *Immunity* 40, 436–450. doi: 10.1016/j.immuni.2014.03.002

EFSA Panel on Dietetic Products, Nutrition and Allergies (NDA) (2012). Scientific opinion on the substantiation of health claims related to various microorganisms and reduction of gastro-intestinal discomfort, decreasing potentially pathogenic gastro-intestinal microorganisms and related claims pursuant to Article 13(1) of Regulation (EC) No 1924/2006. *EFSA J.* 10:2858. doi: 10.2903/j.efsa.2012.2717

EFSA Panel on Nutrition, Novel Foods and Food Allergens (NDA) (2021). Safety of pasteurised *Akkermansia muciniphila* as a novel food. *EFSA J.* 19:e06780. doi: 10.2903/j.efsa.2021.6780

European Medicines Agency (2010). *Guideline on the Investigation of Bioequivalence.* CPMP/EWP/QWP/1401/98 Rev. 1. London: European Medicines Agency.

Evrard, B., Coudeyras, S., Dosgilbert, A., Charbonnel, N., Alamé, J., Tridon, A., et al. (2011). Dose-dependent immunomodulation of human dendritic cells by the probiotic *Lactobacillus rhamnosus* Lcr35. *PLoS One* 6:e18735. doi: 10.1371/journal.pone.0018735

FAO/WHO (2001). *Guidelines for the Evaluation of Probiotics in Food. Report of a Joint FAO/WHO Working Group.* London, Ontario: Food and Agriculture Organization of the United Nations and World Health Organization.

Foglia, C., and Allesina, S. (2020). New insights in enumeration methodologies of probiotic cells in finished products. *J. Microbiol. Methods* 175:105993. doi: 10.1016/j.mimet.2020.105993

Foligné, B., Nutten, S., Grangette, C., Dennin, V., Goudercourt, D., Poiret, S., et al. (2007). Correlation between in vitro and in vivo immunomodulatory properties of lactic acid bacteria. *World J. Gastroenterol.* 13, 236–243. doi: 10.3748/wjg.v13.i2.236

Ghadimi, D., Fölster-Holst, R., de Vrese, M., Winkler, P., Heller, K. J., and Schrezenmeir, J. (2008). Effects of probiotic bacteria and their genomic DNA on TH1/TH2-cytokine production by peripheral blood mononuclear cells (PBMCs) of healthy and allergic subjects. *Immunobiology* 213, 677–692. doi: 10.1016/j.imbio.2008.02.001

Gorsuch, J. P., and Woodruff, P. (2019). Nutrient germination improves DNA recovery from industrial *Bacillus subtilis* endospores during qPCR enumeration assays. *Heliyon* 5:e02917. doi: 10.1016/j.heliyon.2019.e02917

Hill, C., Guarner, F., Reid, G., Gibson, G. R., Merenstein, D. J., Pot, B., et al. (2014). The International Scientific Association for Probiotics and Prebiotics consensus statement on the scope and appropriate use of the term probiotic. *Nat. Rev. Gastroenterol. Hepatol.* 11, 506–514. doi: 10.1038/nrgastro.2014.66

Hong, W., and Zhou, X. (2022). A comparison of XGBoost, random forest, and nomograph for the prediction of disease severity in patients with COVID-19 pneumonia: implications of cytokine and immune cell profile. *Front. Cell. Infect. Microbiol.* 12:819267. doi: 10.3389/fcimb.2022.819267

Hua, M. C., Lin, T. Y., Lai, M. W., Kong, M. S., Chang, H. J., and Chen, C. C. (2010). Probiotic Bio-Three induces Th1 and anti-inflammatory effects in PBMC and dendritic cells. *World J. Gastroenterol.* 16, 3529–3540. doi: 10.3748/wjg.v16.i28.3529

Ingber, D. E. (2022). Human organs-on-chips for disease modelling, drug development and personalized medicine. *Nat. Rev. Genet.* 23, 467–491. doi: 10.1038/s41576-022-00466-9

International Organization for Standardization (2015). *ISO 19344:2015. Milk and Milk Products — Starter Cultures, Probiotics and Fermented Products — Quantification of Lactic Acid Bacteria by Flow Cytometry.* Geneva: International Organization for Standardization.

Iwasaki, A., and Medzhitov, R. (2015). Control of adaptive immunity by the innate immune system. *Nat. Immunol.* 16, 343–353. doi: 10.1038/ni.3123

Klaenhammer, T. R., Kleerebezem, M., Kopp, M. V., and Rescigno, M. (2012). The impact of probiotics and prebiotics on the immune system. *Nat. Rev. Immunol.* 12, 728–734. doi: 10.1038/nri3312

Kleiveland, C. R. (2015). “Peripheral blood mononuclear cells,” in *The Impact of Food Bioactives on Health: In Vitro and Ex Vivo Models*, eds K. Verhoeckx, P. Cotter, I. Lopez-Exposito, C. Kleiveland, T. Lea, A. Mackie, et al. (Cham: Springer), 161–167. doi: 10.1007/978-3-319-16104-4_15

Kristensen, N. B., Bryrup, T., Allin, K. H., Nielsen, T., Hansen, T. H., and Pedersen, O. (2016). Alterations in fecal microbiota composition by probiotic supplementation in healthy adults. *Genome Med.* 8:52. doi: 10.1186/s13073-016-0300-5

Li, P., and Li, M. (2025). Best practices for developing microbiome-based disease diagnostic classifiers through machine learning. *Gut Microbes* 17:2489074. doi: 10.1080/19490976.2025.2489074

Li, Y., Oosting, M., Smeekens, S. P., Jaeger, M., Aguirre-Gamboa, R., Le, K. T. T., et al. (2016). A functional genomics approach to understand variation in cytokine production in humans. *Cell* 167, 1099–1110. doi: 10.1016/j.cell.2016.10.017

Maecker, H. T., McCoy, J. P., and Nussenblatt, R. (2012). Standardizing immunophenotyping for the Human Immunology Project. *Nat. Rev. Immunol.* 12, 191–200. doi: 10.1038/nri3158

McFarland, L. V., Hecht, G., Sanders, M. E., Goff, D. A., Goldstein, E. J. C., Hill, C., et al. (2023). Recommendations to improve quality of probiotic systematic reviews with meta-analyses. *JAMA Netw. Open* 6:e2346872. doi: 10.1001/jamanetworkopen.2023.46872

Meijerink, M., van Hemert, S., Taverne, N., Wels, M., de Vos, P., Bron, P. A., et al. (2010). Identification of genetic loci in Lactobacillus plantarum that modulate the immune response of dendritic cells using comparative genome hybridisation. *PLoS One* 5:e10632. doi: 10.1371/journal.pone.0010632

Neal, J. T., Li, X., Zhu, J., Giangarra, V., Grzeskowiak, C. L., Ju, J., et al. (2018). Organoid modeling of the tumor immune microenvironment. *Cell* 175, 1972–1988. doi: 10.1016/j.cell.2018.11.021

Niers, L. E. M., Timmerman, H. M., Rijkers, G. T., van Bleek, G. M., van Uden, N. O. P., Knol, E. F., et al. (2005). Identification of strong interleukin-10 inducing lactic acid bacteria which down-regulate T helper type 2 cytokines. *Clin. Exp. Allergy* 35, 1481–1489. doi: 10.1111/j.1365-2222.2005.02375.x

Niers, L., Martín, R., Rijkers, G., Sengers, F., Timmerman, H., van Uden, N., et al. (2009). The effects of selected probiotic strains on the development of eczema (the PandA study). *Allergy* 64, 1349–1358. doi: 10.1111/j.1398-9995.2009.02021.x

O’Mahony, L., McCarthy, J., Kelly, P., Hurley, G., Luo, F., Chen, K., et al. (2005). Lactobacillus and bifidobacterium in irritable bowel syndrome: symptom responses and relationship to cytokine profiles. *Gastroenterology* 128, 541–551. doi: 10.1053/j.gastro.2004.11.050

Orkkatteri Krishnan, A., Mudgal, L. N., Soni, V., and Prakash, T. (2025). ProbML: a machine learning-based genome classifier for identifying probiotic organisms. *Mol. Nutr. Food Res.* 69:e70025. doi: 10.1002/mnfr.70025

Ouwehand, A. C. (2017). A review of dose-responses of probiotics in human studies. *Benef. Microbes* 8, 143–151. doi: 10.3920/BM2016.0140

Papadimitriou, K., Zoumpopoulou, G., Foligné, B., Alexandraki, V., Kazou, M., Pot, B., et al. (2015). Discovering probiotic microorganisms: in vitro, in vivo, genetic and omics approaches. *Front. Microbiol.* 6:58. doi: 10.3389/fmicb.2015.00058

Rath, C., Athalye-Jape, G., Rao, S., and Patole, S. (2023). Effect of probiotic dose escalation on gut microbiota and clinical outcomes in preterm infants—a systematic review. *Children (Basel)* 10:1710. doi: 10.3390/children10101710

Salminen, S., Collado, M. C., Endo, A., Hill, C., Lebeer, S., Quigley, E. M. M., et al. (2021). The International Scientific Association of Probiotics and Prebiotics (ISAPP) consensus statement on the definition and scope of postbiotics. *Nat. Rev. Gastroenterol. Hepatol.* 18, 649–667. doi: 10.1038/s41575-021-00440-6

Sato, T., Vries, R. G., Snippert, H. J., van de Wetering, M., Barker, N., Stange, D. E., et al. (2009). Single Lgr5 stem cells build crypt-villus structures in vitro without a mesenchymal niche. *Nature* 459, 262–265. doi: 10.1038/nature07935

Shane, A. L., Cabana, M. D., Vidry, S., Merenstein, D., Hummelen, R., Ellis, C. L., et al. (2010). Guide to designing, conducting, publishing and communicating results of clinical studies involving probiotic applications in human participants. *Gut Microbes* 1, 243–253. doi: 10.4161/gmic.1.4.12707

Suez, J., Segal, E., and Elinav, E. (2019). The pros, cons, and many unknowns of probiotics. *Nat. Med.* 25, 716–729. doi: 10.1038/s41591-019-0439-x

Suez, J., Zmora, N., Zilberman-Schapira, G., Mor, U., Dori-Bachash, M., Bashiardes, S., et al. (2018). Post-antibiotic gut mucosal microbiome reconstitution is impaired by probiotics. *Cell* 174, 1406–1423. doi: 10.1016/j.cell.2018.08.047

Sun, Y., Li, H., Zheng, L., Li, J., Hong, Y., Liang, P., et al. (2022). iProbiotics: a machine learning platform for rapid identification of probiotic properties from whole-genome primary sequences. *Brief. Bioinform.* 23:bbab477. doi: 10.1093/bib/bbab477

Szajewska, H., Scott, K. P., de Meij, T., Forslund-Startceva, S. K., Knight, R., Koren, O., et al. (2025). Antibiotic-perturbed microbiota and the role of probiotics. *Nat. Rev. Gastroenterol. Hepatol.* 22, 155–172. doi: 10.1038/s41575-024-01023-x

Taverniti, V., and Guglielmetti, S. (2011). The immunomodulatory properties of probiotic microorganisms beyond their viability. *Genes Nutr.* 6, 261–274. doi: 10.1007/s12263-011-0218-x

Tedesco, S., De Majo, F., Kim, J., Trenti, A., Trevisi, L., Fadini, G. P., et al. (2018). Convenience versus biological significance: are PMA-differentiated THP-1 cells a reliable substitute for blood-derived macrophages when studying in vitro polarization? *Front. Pharmacol.* 9:71. doi: 10.3389/fphar.2018.00071

Tracey, H., Coates, N., Hulme, E., John, D., Michael, D. R., and Plummer, S. F. (2023). Insights into the enumeration of mixtures of probiotic bacteria by flow cytometry. *BMC Microbiol.* 23:48. doi: 10.1186/s12866-023-02792-2

Trinchieri, G. (2003). Interleukin-12 and the regulation of innate resistance and adaptive immunity. *Nat. Rev. Immunol.* 3, 133–146. doi: 10.1038/nri1001

van Baarlen, P., Troost, F., van der Meer, C., Hooiveld, G., Boekschoten, M., Brummer, R. J., et al. (2011). Human mucosal in vivo transcriptome responses to three lactobacilli indicate how probiotics may modulate human cellular pathways. *Proc. Natl. Acad. Sci. U.S.A.* 108 (Suppl. 1), 4562–4569. doi: 10.1073/pnas.1000079107

van Hemert, S., Meijerink, M., Molenaar, D., Bron, P. A., de Vos, P., Kleerebezem, M., et al. (2010). Identification of Lactobacillus plantarum genes modulating the cytokine response of human peripheral blood mononuclear cells. *BMC Microbiol.* 10:293. doi: 10.1186/1471-2180-10-293

Visciglia, A., Allesina, S., Amoruso, A., De Prisco, A., Dhir, R., Bron, P. A., et al. (2022). Assessment of shelf-life and metabolic viability of a multi-strain synbiotic using standard and innovative enumeration technologies. *Front. Microbiol.* 13:989563. doi: 10.3389/fmicb.2022.989563

Vivier, E., Raulet, D. H., Moretta, A., Caligiuri, M. A., Zitvogel, L., Lanier, L. L., et al. (2011). Innate or adaptive immunity? The example of natural killer cells. *Science* 331, 44–49. doi: 10.1126/science.1198687

Wang, X.-W., and Liu, Y.-Y. (2020). Comparative study of classifiers for human microbiome data. *Med. Microecol.* 4:100013. doi: 10.1016/j.medmic.2020.100013

World Gastroenterology Organisation (2023). *World Gastroenterology Organisation Global Guidelines: Probiotics and Prebiotics.* Milwaukee, WI: World Gastroenterology Organisation.

Wu, J., Zhang, B., Liu, X., Gu, W., Xu, F., Wang, J., et al. (2024). An intelligent intestine-on-a-chip for rapid screening of probiotics with relief-enteritis function. *Adv. Mater.* 36:e2408485. doi: 10.1002/adma.202408485

Wu, S., Feng, T., Tang, W., Qi, C., Gao, J., He, X., et al. (2024). metaProbiotics: a tool for mining probiotic from metagenomic binning data based on a language model. *Brief. Bioinform.* 25:bbae085. doi: 10.1093/bib/bbae085

Zmora, N., Zilberman-Schapira, G., Suez, J., Mor, U., Dori-Bachash, M., Bashiardes, S., et al. (2018). Personalized gut mucosal colonization resistance to empiric probiotics. *Cell* 174, 1388–1405. doi: 10.1016/j.cell.2018.08.041

Zorzela, L., Ardestani, S. K., McFarland, L. V., and Vohra, S. (2017). Is there a role for modified probiotics as beneficial microbes: a systematic review of the literature. *Benef. Microbes* 8, 739–754. doi: 10.3920/BM2017.0032
